# Estimating the Hospital Burden of Norovirus-Associated Gastroenteritis in England and Its Opportunity Costs for Nonadmitted Patients

**DOI:** 10.1093/cid/ciy167

**Published:** 2018-02-26

**Authors:** Frank G Sandmann, Laura Shallcross, Natalie Adams, David J Allen, Pietro G Coen, Annette Jeanes, Zisis Kozlakidis, Lesley Larkin, Fatima Wurie, Julie V Robotham, Mark Jit, Sarah R Deeny

**Affiliations:** 1 Department of Infectious Disease Epidemiology, London School of Hygiene and Tropical Medicine, Public Health England; 2 Statistics, Modelling and Economics Department, National Infection Service, Public Health England (PHE); 3 Department of Infectious Disease Informatics, Institute of Health Informatics, University College London, PHE; 4 Gastrointestinal Infections Department, National Infection Service, PHE; 5 National Institute for Health Research Health Protection Research Unit in Gastrointestinal Infections, PHE; 6 Department of Pathogen Molecular Biology, London School of Hygiene and Tropical Medicine, PHE; 7 Virus Reference Department, National Infection Service, PHE; 8 Infection Control Office, University College Hospitals London, London, United Kingdom; 9 Infection Control Department, University College London Hospitals Trust, London, United Kingdom; 10 Division of Infection and Immunity, University College London, London, United Kingdom; 11 The Health Foundation, London, United Kingdom

**Keywords:** burden of disease, opportunity costs, gastroenteritis, norovirus, outbreaks

## Abstract

**Background:**

Norovirus places a substantial burden on healthcare systems, arising from infected patients, disease outbreaks, beds kept unoccupied for infection control, and staff absences due to infection. In settings with high rates of bed occupancy, opportunity costs arise from patients who cannot be admitted due to beds being unavailable. With several treatments and vaccines against norovirus in development, quantifying the expected economic burden is timely.

**Methods:**

The number of inpatients with norovirus-associated gastroenteritis in England was modeled using infectious and noninfectious gastrointestinal Hospital Episode Statistics codes and laboratory reports of gastrointestinal pathogens collected at Public Health England. The excess length of stay from norovirus was estimated with a multistate model and local outbreak data. Unoccupied bed-days and staff absences were estimated from national outbreak surveillance. The burden was valued conventionally using accounting expenditures and wages, which we contrasted to the opportunity costs from forgone patients using a novel methodology.

**Results:**

Between July 2013 and June 2016, 17.7% (95% confidence interval [CI], 15.6%‒21.6%) of primary and 23.8% (95% CI, 20.6%‒29.9%) of secondary gastrointestinal diagnoses were norovirus attributable. Annually, the estimated median 290000 (interquartile range, 282000‒297000) occupied and unoccupied bed-days used for norovirus displaced 57800 patients. Conventional costs for the National Health Service reached £107.6 million; the economic burden approximated to £297.7 million and a loss of 6300 quality-adjusted life-years annually.

**Conclusions:**

In England, norovirus is now the second-largest contributor of the gastrointestinal hospital burden. With the projected impact being greater than previously estimated, improved capture of relevant opportunity costs seems imperative for diseases such as norovirus.

## Teaser

Since the introduction of rotavirus vaccination in England in July 2013, norovirus has become the second-largest contributor of inpatient gastroenteritis, preventing 57800 patients from being admitted annually. Economic costs amount to £297.7 million, which translates into 6300 quality-adjusted life years.

Norovirus has been associated with almost one-fifth of cases of all-cause acute gastroenteritis worldwide [1], resulting in an estimated median of 698.8 million illnesses and 218800 deaths annually across all ages [2]. Norovirus most commonly occurs in the community [2–4]. However, local hospital outbreaks of norovirus are highly disruptive and have significant economic costs internationally [5–8]. These outbreaks can lead to increased norovirus-specific infections that may reduce available beds within the hospital system through infected patients blocking space for new admissions, beds left unoccupied for reasons of infection control and to allow cleaning and decontamination after outbreaks, and staff absences due to infection [5, 9, 10].

The impact of norovirus on the hospital system prompted the introduction of the English Hospital Norovirus Outbreak Reporting System (HNORS) in 2009 [11], and in 2010 the National Health Service (NHS) England started monitoring the performance of all acute care hospitals during winter [12]. While both systems enable detection of hospital bed pressures and norovirus outbreaks, neither collects individual-patient data. Therefore, the data collected by such surveillance systems alone do not capture the full burden of norovirus. With several antiviral treatments and vaccine candidates in development [13, 14], obtaining a comprehensive overview of the baseline burden of norovirus in hospitals is timely to inform policy makers and investment in control strategies.

Moreover, as acute hospitals in many countries [15], including England [16], face high occupancy rates of beds, patients who cannot be admitted due to beds being unavailable result in health and economic losses to the healthcare system. Costing the burden of hospital infections such as norovirus has previously only considered actual expenditures incurred from dealing with an outbreak [5, 9], ignoring the wider health impact for other patients awaiting admission [17]. This is likely to underestimate the impact of norovirus on the healthcare systems and, consequently, any benefits from investing in novel vaccines, treatments, or infection control.

## METHODS

### Data Sources

#### Number of patients, bed-days lost, and staff absences during norovirus outbreaks

Since 2009, hospitals have been encouraged to voluntarily report norovirus outbreaks (defined as ≥2 cases in a functional care unit) to HNORS at http://bioinformatics.phe.org.uk/noroOBK. Previously, the underreporting in this web-based surveillance system was estimated at about 20% [11]. The numbers of patients, staff absences, and lost bed-days due to norovirus were obtained for all outbreaks declared between July 2009 and by week 27 of June 2016.

### Hospital Statistics for Gastrointestinal Illnesses

The observed number of inpatients with primary and secondary gastrointestinal disease diagnoses and the bed-days occupied by the inpatients with primary diagnoses were obtained for July 2009 to June 2016 from the Hospital Episode Statistics database, which holds all records of NHS hospitalizations in England [18]. Primary diagnoses describe the main reason for hospitalization, whereas secondary diagnoses describe comorbidities of patients treated for another primary medical reason. Cases with all-cause gastroenteritis were identified using the *International Classification of Diseases, Tenth Revision,* and the diagnosis codes of infectious as well as noninfectious intestinal diseases A00‒A09, K52.8, and K52.9 [19, 20].

### Laboratory Data of Gastrointestinal Pathogens

The weekly number of laboratory reports submitted to Public Health England for surveillance purposes by microbiology laboratories across England were obtained for July 2009 to June 2016 for the following gastrointestinal pathogens: adenovirus (enteric infections–associated group F serotypes 40 and 41), astrovirus, *Campylobacter*, *Cryptosporidium*, *Giardia*, norovirus, rotavirus, nontyphoidal *Salmonella* (ie, excluding *Salmonella* Typhi and *Salmonella* Paratyphi), and *Shigella*. *Listeria* cases were obtained from national surveillance of listeriosis in England and Wales. In a separate analysis, cases of Shiga toxin–producing *Escherichia coli* (STEC) were also included as this was only available up to December 2015.

### Patient-Level Data of Norovirus Infections From a Local Hospital

This study obtained individual-level data collected during a norovirus outbreak on 4 wards of a large teaching hospital in London in 2015. Routinely collected data were also obtained on age, sex, dates of admission and discharge, primary and up to 11 secondary diagnosis codes, norovirus sample collection date, and discharge status for all patients admitted to the same wards and days for 2015 with a 2-year look-back. Cases were identified based on the primary diagnosis code, the first positive norovirus genogroup II (GII) infection sample during the hospital stay, and, for the outbreak in 2015, symptom onset.

### Number of Bed-Days Kept Unoccupied due to Norovirus-Like Symptoms During Winters

It is mandatory for acute care hospitals to report the number of bed-days kept unoccupied due to diarrhea and vomiting/norovirus-like symptoms during winters to NHS England since 2010. We obtained these data for winters 2010–2011 to 2015–2016 [12].

For more details on data sources and information retrieval, see Supplementary Material A.

### Statistical Analysis to Estimate the Burden of Disease

#### Linear Regression Models

We used multiple linear regression models to attribute norovirus to patients with gastroenteritis by using the laboratory reports of relevant gastrointestinal pathogens as explanatory variables and inpatients diagnosed with gastrointestinal illnesses as the response variable (Supplementary Material B). In a separate analysis, we limited the data up to December 2015 to be able to include STEC.

### Multistate Models

We estimated the excess length of hospital stays due to norovirus with a multistate model that consisted of 4 mutually exclusive states: (1) admitted (uninfected); (2) infected/diseased; (3) discharged alive; and (4) in-hospital death (Supplementary Material C). After admission (1), all inpatients were discharged alive (3) or died (4); becoming infected/diseased (2) was optional before being discharged alive (3) or dead (4). The model used the empirical transition matrix of inpatients from the local patient-level hospital data. We ran the model separately with all norovirus cases, and for cases with a secondary norovirus diagnosis.

### Adjustments for Potential Underreporting of Unoccupied Bed-Days and Staff Absences

We estimated the number of bed-days kept unoccupied during norovirus outbreaks based on the national surveillance data. As these data are voluntarily reported, there could be underreporting of outbreaks, or lost bed-days. We accounted for underreporting of lost bed-days during an outbreak (and implicitly underreported outbreaks), using the recorded number of unoccupied bed-days mandatorily reported to NHS England (Supplementary Material D). We adjusted the reported number of staff absences by the estimated underreporting of outbreaks [11] and by using a previous norovirus outbreak study in England [5] (Supplementary Material E).

All analyses were performed in R software version 3.3.1 [21]. For the multistate model, we used the R-packages *mvna* to model the hazards between states [22] and *etm* to estimate the excess length of stay [23]. We report the median and interquartile range (IQR) across seasons; for results per season, see Supplementary Material A and H.

### Costing the Burden of Disease

#### Costing Convention

For inpatients with norovirus-associated gastroenteritis and bed-days kept unoccupied, we calculated expenditures conventionally using national administrative accounting data for 2015–2016 [24] (Supplementary Material F). Staff absences due to infection were costed based on the national average wage of nurses in 2015–2016 [25]. To indicate financial (monetary) savings on nonfixed hospital resources if all norovirus cases were to be averted [26], we assumed a proportion of variable costs of 15% of the total healthcare expenditure on norovirus, including staff absence costs [27].

### Opportunity Costing From Forgone Admissions

Given the high occupancy rates of hospital beds in England [16], opportunity costs arise from alternative patients who cannot be admitted due to beds being unavailable [17]. The forgone health gain from hospital treatment was estimated in terms of quality-adjusted life years (QALYs), using our local patient sample (Supplementary Material G). QALY gains beyond 1 year were discounted at 3.5%, and we considered £20000 as monetary value assigned to each QALY gained [28] to calculate net benefits. In case a higher net benefit was achievable with the alternative patients forgone, the sum of the incurred expenditure and the forgone net benefit approximate to opportunity costs [17].

### Sensitivity Analysis

We performed multivariate sensitivity analyses on all input parameters (Supplementary Material F).

### Ethics Approval

Ethical approvals for this study were received from the Ethics Committee of the London School of Hygiene and Tropical Medicine (reference number 11824) and the North West–Liverpool Central Research Ethics Committee (reference 14/NW/1433).

## RESULTS

### Description of the Data

**Figure 1.**
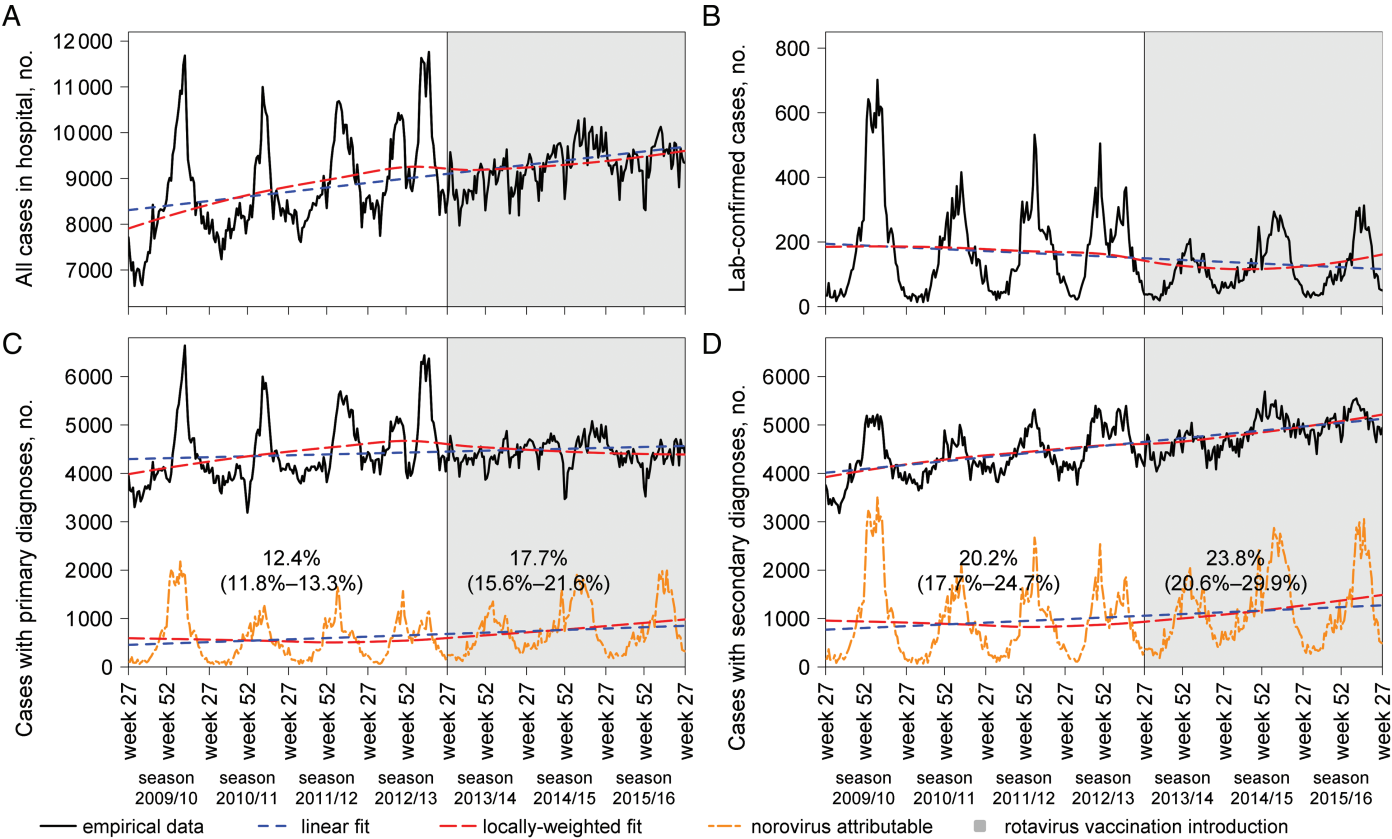
National hospital statistics for inpatients with infectious and noninfectious gastrointestinal (primary and secondary) diagnoses and laboratory-confirmed cases of norovirus in England, July 2009 to June 2016, visualizing norovirus-attributable proportions using linear regressions fitted to the data before and after July 2013. Reported values are the mean and 95% confidence interval (in brackets).

During July 2009 to June 2016, there were a total of 8140 norovirus outbreaks voluntarily reported to HNORS, involving 77800 patients, 20100 staff recorded absent, and 99200 lost bed-days (Supplementary Material A). Of the 658100 enteric laboratory reports in total to national surveillance, the 3 most frequently reported pathogens were *Campylobacter* with 60.7%, rotavirus with 10.8%, and norovirus with 8.6%. Concurrently, Hospital Episode Statistics recorded across all ages 1621000 primary all-cause gastrointestinal diagnoses vs 1672000 patients with secondary all-cause gastrointestinal diagnoses (including 13.1% day cases). The number of primary gastrointestinal diagnoses stabilized after July 2013, whereas the number of patients with secondary gastrointestinal diagnoses kept increasing (Figure 1), driven by infections in adults and the elderly (Supplementary Material A). Based on the data of NHS England, an estimated 142100‒186000 unoccupied acute care hospital bed-days were closed (ie, unavailable) due to norovirus-like symptoms during the 6 winters from 2010–2011 to 2015–2016.

The analysis of the local patient-level sample comprised 2509 individual hospital stays, including 33 associated with norovirus and another 11 with primary infectious intestinal diagnoses (Table 1).

**Table 1.** Demographic Characteristics of the Local Sample of Patients From a Teaching Hospital in London, England, on the Wards Affected by the Norovirus Outbreak of 31 May–15 June 2015, and the Previous 2 Years

Variable	All Patients Analyzed (Cases and Controls)	Control Patients Without Gastroenteritis			Cases With Norovirus		
		Patients With Acute Life-Threatening Conditions^a^	Patients With Chronic Conditions^b^	Patients Without Chronic or Life- Threatening Conditions^c^	Suspected/Confirmed Norovirus Infection^d^	Cases With Primary IID or Norovirus Diagnosis^e^	Cases With Secondary Norovirus Diagnoses
Patients	2509 (88.0)	537 (18.8)	871 (30.5)	1057 (37.0)	33 (1.2)	17 (0.6)	27 (0.9)
Age, y, mean (SD)	59.2 (20.2)	75.1 (14.3)	61.7 (17.6)	48.7 (18.5)	70.8 (18.2)	56.9 (23.5)	73.5 (16.6)
Sex, female	1265 (50.4)	258 (48.0)	422 (48.4)	557 (52.7)	21 (63.6)	11 (64.7)	17 (63.0)
CCI score (>0)	1440 (57.4)	537 (100.0)	871 (100.0)	0 (0.0)	27 (81.8)	8 (47.1)	24 (88.9)
In-hospital mortality	54 (2.2)	21 (3.9)	26 (3.0)	*	*	0 (0.0)	*
LOS, d, mean (range)	5.0 (0–43)	7.2 (0–43)	5.4 (0–43)	3.3 (0–40)	15.8 (3–43)	5.7 (0–25)	17.0 (3–43)
Excess LOS, d, mean (95% CI)^f^	NA	NA	NA	NA	3.33 (.17–6.50)	NA	3.95 (.35–7.55)
QALY gain (undiscounted), mean (95% CI)^g^	0.179 (.0001–.386)	0.307 (.175–.377)	0.313 (.189–.403)	0.002 (.00005–.017)	0.227 (.003–.358)	0.102 (.0004–.295)	0.250 (.009–.365)
QALY gain (discounted), mean (95% CI)^g^	0.142 (.0001–.293)	0.260 (.162–.309)	0.239 (.175–.308)	0.002 (.00005–.017)	0.188 (.003–.295)	0.078 (.0004–.190)	0.211 (.009–.299)

Data are presented as No. (%) unless otherwise indicated. The asterisk (*) indicates a figure between 1 and 5, values suppressed to prevent possible identification of individuals [18].

Abbreviations: CCI, Charlson comorbidity index; CI, confidence interval; GII, norovirus genogroup II; IID, infectious intestinal disease; LOS, length of stay; NA, not applicable; PCR, polymerase chain reaction; QALY, quality-adjusted life-year; SD, standard deviation.

^a^Myocardial infarction, congestive heart failure, or cerebrovascular disease.

^b^CCI > 0 but not acutely life-threatening (ie, myocardial infarction, congestive heart failure, or cerebrovascular disease).

^c^CCI = 0, ie, no chronic or life-threatening conditions.

^d^Suspected infection (for the norovirus outbreak cluster in 2015) and all laboratory-confirmed norovirus GII infections; partly overlapping.

^e^Patients with a primary gastrointestinal diagnosis and laboratory-confirmed norovirus infection (n = 6) or without confirmed norovirus infection (n = 11). No excess LOS is presented here given that hospitalizations for a primary IID but without laboratory-confirmed norovirus diagnosis cannot necessarily be categorized as an excess stay.

^f^Estimated with the multistate model (Supplementary Material C).

^g^For cases with secondary norovirus diagnoses, the QALYs gained were driven by the high level of comorbidities. If we approximated the gastroenteritis-related health gain by subtracting the QALY gain of control patients from the QALY gain of inpatients with secondary norovirus diagnoses, we derive 0.211 – 0.142 = 0.069 (ie, close to the gain of primary cases). For all cases, the activity-weighted mean (discounted) QALY gain amounted to (0.069 × 27 + 0.078 × 17) / 44 = 0.072 QALYs gained, ie, about half of the control patients who gained 0.142 QALYs (see Supplementary Table 4).

### Statistical Analysis to Estimate the Burden of Disease

#### Linear Regression Model Results

**Figure 2.**
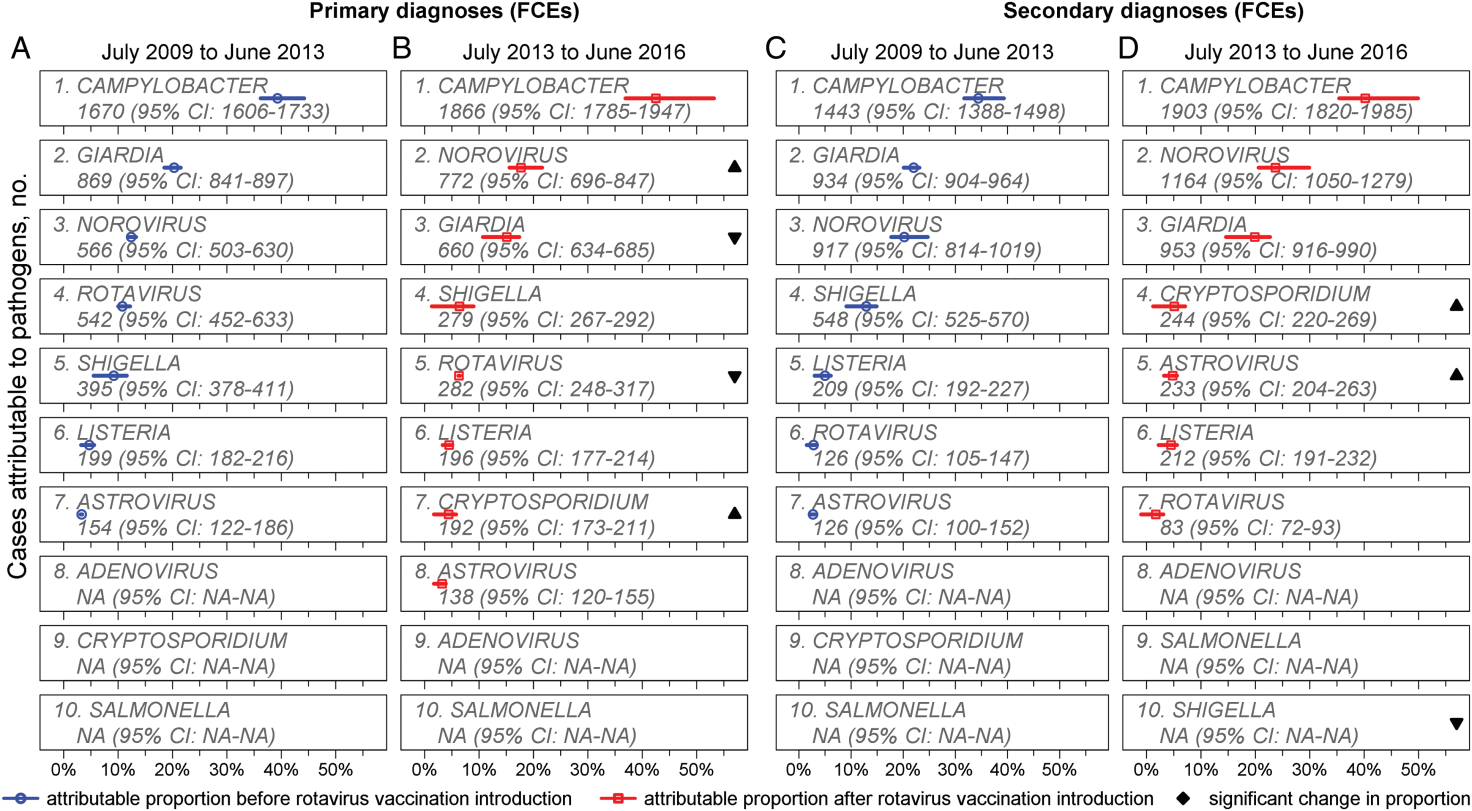
Attributable fraction (%) of enteric pathogens on all-cause acute gastrointestinal primary and secondary diagnoses in hospitals in England, using linear regressions fitted to the data of July 2009 to June 2013 vs July 2013 to June 2016. Estimated absolute numbers provided for information. Abbreviations: CI, confidence interval; FCE, finished consultant episode; NA, not applicable.

The regression models with the highest goodness-of-fit showed a significantly increasing proportion of primary and all diagnoses being attributable to norovirus after July 2013, whereas the proportions of rotavirus-attributable primary and all diagnoses decreased significantly after July 2013 (Supplementary Material B and Figure 2). Due to the heterogeneity across the 7 seasons, we continue reporting results for July 2013 to June 2016. Moreover, the significant reduction in rotavirus diagnoses was driven by 0- to 4-year-olds, while the significant increase for norovirus was driven by patients aged 0–64 years (Supplementary Material B). Given that confidence intervals (CIs) for norovirus overlapped across age groups, we continue reporting nonstratified results.

Between July 2013 and June 2016, the best-fitting regression models attributed 17.7% of primary gastrointestinal diagnoses (95% CI, 15.6%‒21.6%) and 23.8% (95% CI, 20.6%‒29.9%) of secondary gastrointestinal diagnoses to norovirus (Figure 1), leading to a median estimate of 40800 (IQR, 40500‒41400) norovirus-associated cases with primary and 61500 (IQR, 58700‒62500) with secondary diagnoses annually. Results were slightly lower when limiting the data to December 2015 to include STEC (Supplementary Material B).

#### Multistate Model Results

The mean excess length of hospital stay due to norovirus was estimated at 3.3 days (95% CI, .2‒6.5 days). Patients with norovirus infection and a secondary gastrointestinal diagnosis stayed an excess 4.0 days (95% CI, .4‒7.6 days).

#### Bed-Days Kept Unoccupied for Infection Control

A median of 19.7%‒26.3% of bed-days lost in outbreaks voluntarily reported to HNORS by acute care hospitals during the winters of 2013–2014 to 2015–2016 matched with those mandatorily recorded by NHS England (Supplementary Material D).

#### Total Number of Bed-Days Used for Norovirus

Annually, at least 290000 (IQR, 282000‒297000) occupied and unoccupied bed-days were norovirus attributable using conservative estimates (Supplementary Material H), with 28% being used by inpatients with a primary diagnosis, 62% by secondary diagnoses, and 10% were beds that had been closed unoccupied.

### Staff Absences due to Norovirus

An estimated median of 4200 (IQR, 3800‒5100) members of staff were absent during norovirus outbreaks annually between mid-2013 and mid-2016.

### Costing the Burden of Disease

#### Direct Expenditures Incurred due to Norovirus

Norovirus-associated gastroenteritis incurred direct expenditures of £107.6 million (IQR, £104.6‒£109.8 million) annually, of which £8.9 million (IQR, £8.6‒£10.4 million) were lost on unoccupied bed-days. Staff absences due to infection incurred costs for the NHS of £1.3 million (IQR, £1.2‒£1.6 million) annually. The 15% variable costs proportion indicates potential monetary savings from averting all norovirus cases equivalent to £16.1 million (IQR, £15.7‒£16.5 million).

#### Opportunity Costing

The 290000 norovirus-associated bed-days could have been used for 57800 (IQR, 56 400‒59200) alternative nongastroenteritis patients, who would have been expected to gain 13800 (IQR, 13500‒14100) QALYs at a net monetary benefit of £190.1 million (IQR, £185.5‒£194.7 million). From a health-maximizing perspective, the forgone nongastroenteritis patients were expected to have gained a higher net benefit than the norovirus cases (Supplementary Material H), with the value of the opportunity costs approximating to £297.7 million (IQR, £290.1‒£304.5 million) and losing an estimated 6300 QALYs (ie, 13800 minus 7500; see Supplementary Material H).

#### Sensitivity Analysis

Sensitivity analyses confirmed that the wide uncertainty range around the excess length of stay estimate was the most influential source of uncertainty for the burden estimation (Supplementary Material I).

For the estimation of the costs, the monetary value assigned to QALYs was the most influential parameter with a direct impact on the estimates of the economic costs (Supplementary Material I).

## DISCUSSION

This study quantified the hospital burden of norovirus-associated gastroenteritis for the NHS in England. It included for the first time the wider health impact that infectious diseases such as norovirus can have for other patients awaiting admission by reducing the beds and staff available to them [17].

### Summary of Key Findings and Clinical Implications

Of all inpatients with primary or secondary all-cause gastrointestinal diagnoses in England between July 2013 and June 2016, 18% (95% CI, 16%‒22%) and 24% (95% CI, 21%‒30%) were attributable to norovirus, respectively. While the general increase in patients diagnosed with gastrointestinal illnesses in England throughout this period seemed to be driven by secondary diagnoses, the increase in norovirus-attributable inpatients identified after July 2013 appeared to be driven by primary diagnoses (less so by secondary diagnoses or outbreaks or coding variations; see Supplementary Material A). Discharging gastrointestinal inpatients faster in recent years (Supplementary Material A) and enhanced hygiene and other infection control measures [10, 29, 30], as well as a potentially increasing awareness [10], may all have contributed to fewer secondary norovirus-associated hospital cases and norovirus outbreaks.

Our regression results also showed that norovirus is now the second-highest contributor of gastrointestinal hospital diagnoses, after *Campylobacter*. The proportional increase in the burden of norovirus is largely driven by the reduction in rotavirus, which led to a reduced total number of laboratory-confirmed cases (see Supplementary Material A). Overall, the total number of bed-days tied up by norovirus-associated gastroenteritis annually is equivalent to the entire daily NHS hospital bed capacity in England being unavailable for >2 days (ie, 290000 / 133000 [16]). The bed-days lost to norovirus prevented admission of other patients, who are estimated to have the potential to gain twice as many QALYs from hospitalization as the norovirus patients who displaced them. Therefore, our findings demonstrate the wider impact of norovirus outbreaks on health. In addition, a combined £10.3 million was lost annually from bed-days kept unoccupied and staff absences. With the prospect of several norovirus treatments and vaccines becoming available soon [13, 14], our estimates may serve as baseline for future analyses.

### Comparison With Previous Work

Our estimate of 16% (95% CI, 15%–19%) of gastrointestinal patients attributable to norovirus for July 2009 to June 2013 is consistent with a previous estimate of 17% (95% CI, 15%‒19%) from a systematic review of studies published up to 2014 [1], irrespective of novel norovirus strain emergences. After July 2013, we estimated that this increases to 21% (95% CI, 19%‒25%), which is not attributable to the emergence of a novel norovirus strain, but appeared to be driven by statistically significantly higher proportions in children aged 0–4 and 5–18 years and adults aged 19–64 years (Figure 2).

Previously, the costs attributable to unavailable bed-days due to norovirus-like symptoms for acute care hospitals were estimated as £35‒£49 million in England each winter using the excess bed-day cost value for 2015–2016 [31]. Another study estimated the hospital costs of gastroenteritis outbreaks in England at £115 million annually using a top-down approach and data from 1994–1995 and 2002–2003 [5], which translates to costs from norovirus outbreaks of £96.9 million in 2016 value [32] when accounting for norovirus being present in only 63% of gastroenteritis outbreaks [5]. While we estimated higher expenditures of £107.6 million, the economic costs of £297.7 million (including hospitalizations forgone) are almost thrice as large.

### Strengths and Limitations

This study is the first to combine individual-level data with national hospital surveillance and statistics to apply a novel method for estimating the opportunity costs of norovirus infections in hospital from patients who cannot be admitted due to beds being unavailable. This novel methodology is generalizable to other settings, given high bed occupancy rates in other places besides England [15, 16]. Moreover, given the interest in the economic value of the bed-days that could not be used for alternative admissions, no actual cases may have always been delayed or canceled. Furthermore, the approach could be applied to community settings using, for example, general practitioner visits instead of hospitalizations [17]. The potential for these analytical approaches is likely to increase in the future given the increasing number of linkable data sources.

Our study here used the best available data sources for norovirus, which we adjusted for bias in reporting [11, 12]. Unadjusted HNORS data were not used directly due to underreporting of outbreaks, cases, and bed-days. Although our regression analysis assumed independence of observations, it is a well-described method to quantify the etiology of gastroenteritis [19, 20, 33] that captures correlations in weekly counts implicitly through the explanatory variables. Given the large sizes of the data used for the regression analysis, a linear model was chosen; however, results are also robust to a negative binomial model (Supplementary Material B). The regression also accounted for potential miscoding of intestinal diagnoses [19, 20], and we used a statistically rigorous method accounting for time-dependent biases [34–36] to estimate the excess length of stay. A possible limitation is our use of the local data to model length of stay and the expected QALY gain from hospital treatment. Moreover, we took the previously estimated 20% underreporting of outbreaks as a conservative estimate; the actual number of outbreaks may be higher. Likewise, the actual number of bed-days lost unoccupied remains uncertain due to the voluntary reporting to HNORS, and is likely higher than we assumed conservatively here despite our efforts of matching the bed-days. Future research should consider larger norovirus samples through advanced individual-patient infection control during outbreaks and longer observation periods.

The need to differentiate inpatients by their primary and secondary norovirus diagnosis arose mainly in order to not bias the total number of bed-days used for norovirus systematically upward [34–36]. Therefore, we relied on records of intestinal illness episodes in hospital; if illnesses are incompletely recorded, our burden estimate may be an underestimate. Moreover, clinical hospital diagnoses may not fully capture patients with asymptomatic carriage of norovirus [37], and secondary diagnosis codes may be less reliable due to potential variation in coding practices between hospitals [18]. Note that a secondary diagnosis does not necessarily imply a hospital-acquired infection. From the economic perspective of this study, however, the source of infection (ie, hospital acquired or community acquired) is irrelevant for obtaining a comprehensive picture of the burden of norovirus in hospitalized patients.

While our local sample did not involve pediatric or elderly wards, these were included in the national sources. We also did not consider indirect costs from productivity losses nor the costs borne by the community (or in long-term care facilities), which would substantially increase costs [2, 3, 38].

Our results support the hypothesis that a norovirus vaccine may have the greatest impact when reaching adult and the elderly populations, particularly those at risk of infection while staying in hospital (Figure 2 and Supplementary Material A). However, future research needs to investigate the most appropriate target groups to prevent transmission, such as adult inpatients, staff, and children [39,40].

## CONCLUSIONS

With bed pressures being a recurring public health concern, any analysis considering the impact of infectious diseases on hospital systems needs to include the opportunity costs from forgone alternative admissions. In England, norovirus has become the second-largest contributor of inpatient gastrointestinal illnesses in England since mid-2013. Norovirus-associated gastroenteritis ties up the equivalent of more than twice the daily hospital bed stock in England, with a substantial economic and health impact for the NHS and patients.

## Supplementary Data

Supplementary materials are available at *Clinical Infectious Diseases* online. Consisting of data provided by the authors to benefit the reader, the posted materials are not copyedited and are the sole responsibility of the authors, so questions or comments should be addressed to the corresponding author.

Supplementary Material

## Notes

***Acknowledgments.*** The authors thank all hospitals in England that have been submitting data to Hospital Norovirus Outbreak Reporting System at Public Health England (PHE) and to National Health Service (NHS) England’s winter situation reports, the microbiological laboratories across England that have been sending data to PHE, and the outbreak investigation team at University College London Hospital. Particular thanks go to Dr Eleni Nastouli for her support during the outbreak investigation. We thank NHS England for the open access to its winter situation reports. We are grateful to Lisa Byrne, Lukeki Kaindama, and Sanch Kanagarajah of PHE for the provision of counts of cases of *Listeria* and Shiga toxin– producing *Escherichia coli* (STEC) from the National Enhanced Surveillance System for STEC (NESSS) and from national surveillance of listeriosis in England and Wales. Hospital Episode Statistics of the Health and Social Care Information Centre, Copyright © 2017, were reused with the permission of the Health and Social Care Information Centre. All rights reserved.

***Disclaimer.*** The views expressed are those of the authors and not necessarily those of the NHS, the National Institute for Health Research (NIHR), the Department of Health, the Wellcome Trust, or PHE.

***Financial support.*** Z. K. was supported by the Health Innovation Challenge Fund grant ICONIC funded by the Department of Health and the Wellcome Trust (grant reference T5-344). F. W. was funded by a small grant from the Royal Free Charity. M. J. was supported by the NIHR Health Protection Research Unit (HPRU) in Immunisation at the London School of Hygiene and Tropical Medicine in partnership with PHE (grant reference code HPRU-2012–10096). N. A. and D. J. A. are affiliated with the NIHR HPRU in Gastrointestinal Infections at University of Liverpool in partnership with PHE, in collaboration with University of East Anglia, University of Oxford, and the Quadram Institute (grant reference code HPRU-2012–10038). N. A. is based at PHE and the University of Liverpool. D. J. A. is based at the London School of Hygiene and Tropical Medicine and affiliated with PHE.

***Potential conflicts of interest.*** D. J. A. gave an invited presentation at an Infectious Disease Research Network meeting on norovirus in October 2014 that was supported by Takeda Pharmaceuticals. All other authors report no potential conflicts. All authors have submitted the ICMJE Form for Disclosure of Potential Conflicts of Interest. Conflicts that the editors consider relevant to the content of the manuscript have been disclosed.

## References

[1] Ahmed SM, Hall AJ, Robinson AE, et al Global prevalence of norovirus in cases of gastroenteritis: a systematic review and meta-analysis. Lancet Infect Dis 2014; 14:725–30. 24981041 10.1016/S1473-3099(14)70767-4 PMC8006533

[2] Bartsch SM, Lopman BA, Ozawa S, Hall AJ, Lee BY Global economic burden of norovirus gastroenteritis. PLoS One 2016; 11:e0151219. 27115736 10.1371/journal.pone.0151219 PMC4846012

[3] O’Brien SJ, Donaldson AL, Iturriza-Gomara M, Tam CC Age-specific incidence rates for norovirus in the community and presenting to primary healthcare facilities in the United Kingdom. J Infect Dis 2016; 213(Suppl 1):S15–8. 26744427 10.1093/infdis/jiv411 PMC4704656

[4] Kambhampati A, Koopmans M, Lopman BA Burden of norovirus in healthcare facilities and strategies for outbreak control. J Hosp Infect 2015; 89:296–301. 25726433 10.1016/j.jhin.2015.01.011 PMC4668703

[5] Lopman BA, Reacher MH, Vipond IB, et al Epidemiology and cost of nosocomial gastroenteritis, Avon, England, 2002–2003. Emerg Infect Dis 2004; 10:1827–34. 15504271 10.3201/eid1010.030941 PMC3323246

[6] Johnston CP, Qiu H, Ticehurst JR, et al Outbreak management and implications of a nosocomial norovirus outbreak. Clin Infect Dis 2007; 45:534–40. 17682985 10.1086/520666

[7] Lopman BA, Hall AJ, Curns AT, Parashar UD Increasing rates of gastroenteritis hospital discharges in US adults and the contribution of norovirus, 1996–2007. Clin Infect Dis 2011; 52:466–74. 21258098 10.1093/cid/ciq163

[8] Morton VK, Thomas MK, McEwen SA Estimated hospitalizations attributed to norovirus and rotavirus infection in Canada, 2006–2010. Epidemiol Infect 2015; 143:3528–37. 25991407 10.1017/S0950268815000734 PMC4657031

[9] Danial J, Cepeda JA, Cameron F, Cloy K, Wishart D, Templeton KE Epidemiology and costs associated with norovirus outbreaks in NHS Lothian, Scotland 2007–2009. J Hosp Infect 2011; 79:354–8. 21955453 10.1016/j.jhin.2011.06.018

[10] Harris JP, Adak GK, O’Brien SJ To close or not to close? Analysis of 4 year’s data from national surveillance of norovirus outbreaks in hospitals in England. BMJ Open 2014; 4:e003919. 10.1136/bmjopen-2013-003919 PMC3902402 24413345

[11] Harris JP, Adams NL, Lopman BA, Allen DJ, Adak GK The development of Web-based surveillance provides new insights into the burden of norovirus outbreaks in hospitals in England. Epidemiol Infect 2014; 142:1590–8. 24230984 10.1017/S0950268813002896 PMC9151232

[12] National Health Service England. Winter daily situation reports Available at: http://www.england.nhs.uk/statistics/statistical-work-areas/winter-daily-sitreps/. Accessed 26 January 2017.

[13] Riddle MS, Walker RI Status of vaccine research and development for norovirus. Vaccine 2016; 34:2895–9. 27036510 10.1016/j.vaccine.2016.03.077

[14] Debbink K, Lindesmith LC, Baric RS The state of norovirus vaccines. Clin Infect Dis 2014; 58:1746–52. 24585561 10.1093/cid/ciu120 PMC4036685

[15] World Health Organization Regional Office for Europe. Bed occupancy rate, acute care hospitals only Available at: https://gateway.euro.who.int/en/visualizations/line-charts/hfa_542-bed-occupancy-rate-acute-care-hospitals-only/. Accessed 6 August 2017.

[16] National Health Service England. Bed availability and occupancy data—overnight Available at: https://www.england.nhs.uk/statistics/statistical-work-areas/bed-availability-and-occupancy/bed-data-overnight/. Accessed 17 February 2017.

[17] Sandmann FG, Robotham JV, Deeny SR, Edmunds WJ, Jit M Estimating the opportunity costs of bed-days. Health Econ 2017. doi:10.1002/hec.3613. 10.1002/hec.3613 PMC5900745 29105894

[18] Health and Social Care Information Centre. Hospital admitted patient care activity, 2015–16 Available at: http://www.content.digital.nhs.uk/catalogue/PUB22378/hosp-epis-stat-admi-summ-rep-2015-16-rep.pdf. Accessed 03 February 2017.

[19] Ryan MJ, Ramsay M, Brown D, Gay NJ, Farrington CP, Wall PG Hospital admissions attributable to rotavirus infection in England and Wales. J Infect Dis 1996; 174(Suppl 1):S12–8. 8752285 10.1093/infdis/174.supplement_1.s12

[20] Harris JP, Jit M, Cooper D, Edmunds WJ Evaluating rotavirus vaccination in England and Wales. Part I: estimating the burden of disease. Vaccine 2007; 25:3962–70. 17395343 10.1016/j.vaccine.2007.02.072

[21] R Core Team. R: A language and environment for statistical computing Available at: https://www.R-project.org/. Accessed 26 January 2017.

[22] Allignol A, Beyersmann J, Schumacher M mvna: an R package for the Nelson-Aalen estimator in multistate models. R News 2008; 8:48–50.

[23] Allignol A, Schumacher M, Beyersmann J Empirical transition matrix of multi-state models: the etm package. J Stat Softw 2011; 38:1–15.

[24] UK Department of Health. NHS reference costs 2015 to 2016 Available at: https://www.gov.uk/government/publications/nhs-reference-costs-2015-to-2016. Accessed 4 February 2017.

[25] Royal College of Nursing. NHS pay scales 2015–16: pay scales for NHS nursing staff in England, Wales, Scotland and Northern Ireland from 1 April 2015 Available at: https://www.rcn.org.uk/employment-and-pay/nhs-pay-scales-2015–16. Accessed 3 February 2017.

[26] Graves N, Harbarth S, Beyersmann J, Barnett A, Halton K, Cooper B Estimating the cost of health care-associated infections: mind your p’s and q’s. Clin Infect Dis 2010; 50:1017–21. 20178419 10.1086/651110

[27] Plowman R, Graves N, Griffin MA, et al The rate and cost of hospital-acquired infections occurring in patients admitted to selected specialties of a district general hospital in England and the national burden imposed. J Hosp Infect 2001; 47:198–209. 11247680 10.1053/jhin.2000.0881

[28] National Institute for Health and Care Excellence. Guide to the methods of technology appraisal 2013. London: NICE, 2013. 27905712

[29] Norovirus Working Party. Guidelines for the management of norovirus outbreaks in acute and community health and social care settings. 2012 Available at: https://www.gov.uk/government/publications/norovirus-managing- outbreaks-in-acute-and-community-health-and-social-care-settings. Accessed 11 June 2017.

[30] Sadique Z, Lopman B, Cooper BS, Edmunds WJ Cost-effectiveness of ward closure to control outbreaks of norovirus infection in United Kingdom National Health Service Hospitals. J Infect Dis 2016; 213(Suppl 1):S19–26. 26744428 10.1093/infdis/jiv410

[31] Sandmann FG, Jit M, Robotham JV, Deeny SR Burden, duration, and costs of hospital bed closures due to acute gastroenteritis in England per winter, 2010/11–2015/16. J Hosp Infect 2017; 7:79–85. 10.1016/j.jhin.2017.05.015 PMC5564405 28552406

[32] Office for National Statistics. CPI All Items Index: estimated pre-97 2015 = 100. Available at: https://www.ons.gov.uk/economy/inflationandpriceindices/timeseries/d7bt/mm23. Accessed 28 June 2017.

[33] Haustein T, Harris JP, Pebody R, Lopman BA Hospital admissions due to norovirus in adult and elderly patients in England. Clin Infect Dis 2009; 49:1890–2. 19911997 10.1086/648440

[34] De Angelis G, Murthy A, Beyersmann J, Harbarth S Estimating the impact of healthcare-associated infections on length of stay and costs. Clin Microbiol Infect 2010; 16:1729–35. 20673257 10.1111/j.1469-0691.2010.03332.x

[35] Beyersmann J, Gastmeier P, Wolkewitz M, Schumacher M An easy mathematical proof showed that time-dependent bias inevitably leads to biased effect estimation. J Clin Epidemiol 2008; 61:1216–21. 18619803 10.1016/j.jclinepi.2008.02.008

[36] Barnett AG, Beyersmann J, Allignol A, Rosenthal VD, Graves N, Wolkewitz M The time-dependent bias and its effect on extra length of stay due to nosocomial infection. Value Health 2011; 14:381–6. 21402305 10.1016/j.jval.2010.09.008

[37] Harris JP, Iturriza-Gomara M, O’Brien SJ Re-assessing the total burden of norovirus circulating in the United Kingdom population. Vaccine 2017; 35:853–5. 28094075 10.1016/j.vaccine.2017.01.009 PMC5287221

[38] Tam CC, O’Brien SJ Economic cost of campylobacter, norovirus and rotavirus disease in the United Kingdom. PLoS One 2016; 11:e0138526. 26828435 10.1371/journal.pone.0138526 PMC4735491

[39] Simmons K, Gambhir M, Leon J, Lopman B Duration of immunity to norovirus gastroenteritis. Emerg Infect Dis 2013; 19:1260–7. 23876612 10.3201/eid1908.130472 PMC3739512

[40] Steele MK, Remais JV, Gambhir M, et al Targeting pediatric versus elderly populations for norovirus vaccines: a model-based analysis of mass vaccination options. Epidemics 2016; 17:42–9. 27821278 10.1016/j.epidem.2016.10.006 PMC5206891

