# Supplementary material for: Estimating the Hospital Burden of Norovirus-Associated Gastroenteritis in England and Its Opportunity Costs for Nonadmitted Patients
Source: Clin Infect Dis. 2018 Feb 26;67(5):693–700. doi: 10.1093/cid/ciy167 (PMC6094002; doi:10.1093/cid/ciy167)
Supplement: Supplementary Material [file ciy167_suppl_supplementary_material.docx]

# Estimating the hospital burden of norovirus-associated gastroenteritis in England and its opportunity costs for non-admitted patients

Frank G. Sandmann^1,2^, Laura Shallcross^3^, Natalie Adams^4,5^, David J. Allen^5,6,7^, Pietro G. Coen^8^, Annette Jeanes^9^, Zisis Kozlakidis^3,10^, Lesley Larkin^4^, Fatima Wurie^3^, Julie V. Robotham^2^, Mark Jit^1,2^, Sarah R. Deeny^11^

## Supplementary Material

A. Additional information on data sources 2

B. Details on the linear regression analysis 8

C. Details on the multi-state model 14

D. Details on the comparison of bed-days kept unoccupied for infection control 17

E. Details on the estimated number of staff being absent during norovirus outbreaks 21

F. Details of the costing approach 22

G. Details on the modelled health gain expected from hospital treatment 24

H. Details on results of the burden and costs estimation 26

I. Details on the sensitivity analysis 29

J. References 31

1. Additional information on data sources

We estimated the hospital burden of norovirus-associated gastroenteritis based on a) patients, b) the bed-days occupied by these patients, c) bed-days kept unoccupied for infection control, and d) staff absences due to infection. While our starting point was the national hospital surveillance system of norovirus outbreaks, HNORS, this system only tracks outbreaks and relies on voluntary reporting. As such, we used HES data to obtain all norovirus-attributable gastroenteritis patients, and we used the voluntarily reported numbers of lost bed-days and staff absences in HNORS as basis for our estimation after adjusting for potential under-reporting.

#### Number of patients, bed-days lost, and staff absences during norovirus outbreaks

The Hospital Norovirus Outbreak Reporting System (HNORS) gathers voluntarily reported information of norovirus outbreaks since its inception in January 2009. In total, HNORS included 8,767 outbreaks at the time of data retrieval (August 2016). We excluded incomplete outbreaks up to June 2009 as they counted towards various previous epidemiological seasons (n=609), and outbreaks for the season of 2016/17 (n=16). In total, 8,142 outbreaks in 439 hospitals and 147 Trusts across the seven seasons from July 2009 to June 2016 were considered in our analysis. Most outbreaks involved general medicine wards (n=1,754; 21.5%), followed by elderly care wards (n=1,587; 19.5%) and acute medicine (n=511; 6.3%). Paediatric wards were only involved in a small number of outbreaks (n=88; 1.1%).

#### Hospital statistics for gastrointestinal illnesses

The Hospital Episode Statistics (HES) database holds inpatient records including one primary and up to 19 secondary diagnoses that are recorded at the time of discharge [1]. Gastrointestinal infectious and non-infectious illnesses (ICD10 codes A00‒A09; K528 and K529) were extracted per date of admission for finished consultant episodes using only primary diagnosis codes, only secondary diagnosis codes, or all diagnosis codes of all ordinary admissions, day cases, and mothers and babies using only delivery facilities. It was ensured that patients with secondary diagnoses were not double counted given that records can have more than one gastrointestinal diagnosis code, and also both a primary plus secondary diagnosis code (which were counted as primary diagnosis).

Given that the HES data are recorded per financial year (i.e., from April in the first year up to the end of March of the following year), we obtained records for 2016/17 too in order to obtain data up to week 26 in 2016 to derive the entire epidemiological season for 2015/16.

Interestingly, a change in coding practices led to an increase in infectious intestinal diagnoses on all gastrointestinal diagnoses from 22.9% to 87.1% starting in financial year 2012/13 (Supplementary Table 2). As such, we combined infectious and non-infectious gastrointestinal diagnoses in the analysis in order to minimise the impact of coding variations over time.

| Table 2: Coding of infectious and non-infectious intestinal diagnoses in England over time, 2009/10–2015/16. | | | | | | | | | |
| --- | --- | --- | --- | --- | --- | --- | --- | --- | --- |
| Season | 2009/10 | 2010/11 | 2011/12 | 2012/13 | 2013/14 | 2014/15 | 2015/16 | **2009/10–2011/12** | **2012/13–2015/16** |
| *Patients with primary gastrointestinal diagnoses* | | | | | | | | | |
| infectious^a^ | 0.312 | 0.300 | 0.288 | 0.859 | 0.869 | 0.863 | 0.857 | 0.300 | 0.861 |
| non-infectious^b^ | 0.688 | 0.700 | 0.712 | 0.141 | 0.131 | 0.137 | 0.143 | 0.700 | 0.139 |
| *Patients with secondary gastrointestinal diagnoses* | | | | | | | | | |
| infectious^a^ | 0.181 | 0.159 | 0.153 | 0.871 | 0.890 | 0.887 | 0.883 | 0.159 | 0.883 |
| non-infectious^b^ | 0.819 | 0.841 | 0.847 | 0.129 | 0.110 | 0.113 | 0.117 | 0.841 | 0.117 |
| *Patients with primary or secondary gastrointestinal diagnoses* | | | | | | | | | |
| infectious^a^ | 0.248 | 0.229 | 0.221 | 0.865 | 0.880 | 0.876 | 0.871 | 0.229 | 0.871 |
| non-infectious^b^ | 0.752 | 0.771 | 0.779 | 0.135 | 0.120 | 0.124 | 0.129 | 0.771 | 0.129 |
| Note: Data represent financial years.  a: ICD-10 codes A00‒A09 across all ages.  b: ICD-10 codes K528 and K529 across all ages. | | | | | | | | | |

#### Hospital statistics for gastrointestinal illnesses: Further dynamics observed

Patients with a primary gastrointestinal diagnosis have used statistically significant fewer bed-days over the years, with a median 2.21 (IQR: 2.21–2.24) bed-days before mid-2013 and 1.99 (IQR: 1.96–1.99) afterwards (Supplementary Table 3).

Moreover, the increase of primary gastrointestinal diagnoses appeared to have been halted for most age groups after introducing the rotavirus vaccine (Supplementary Figure 3); the further increase for adolescents seemed minor given the low number of cases. More importantly, the very high peaks for young children (aged <5 years) disappeared after July 2013, which included the primary target group of the rotavirus vaccination campaign [2]. Unlike primary diagnoses, the number of inpatients with secondary gastrointestinal diagnoses continued to increase across all ages except for young children (aged < 5 years), whose peaks also flattened slightly (Supplementary Figure 3). However, winter peaks were still visible for young children (aged <5 years) and the elderly.

Consequently, in health care settings the major gastrointestinal disease burden now rests with adults and the elderly since July 2013, particularly for patients with secondary gastrointestinal diagnoses (cf. Supplementary Table 3 and Supplementary Figure 3; note the different scales in Supplementary Figure 3). While our local sample did not involve paediatric or elderly wards, these were included in the national sources. Future research may need to investigate norovirus transmission rates for different age groups. When stratifying regressions by age, confidence intervals overlapped, which supports a previous systematic review and meta-analysis that found similar norovirus attributable fractions among all-cause gastroenteritis for patients below the age of 5, 5 years and older, and mixed ages [1].

| Table 3. Raw input data of the national data sources in England per season, for NHSE per winter. | | | | | | | | | |
| --- | --- | --- | --- | --- | --- | --- | --- | --- | --- |
| Source | Variable | 2009/10 | 2010/11 | 2011/12 | 2012/13 | 2013/14 | 2014/15 | 2015/16 |  |
| SGSS/NESSS/national surveillance of listeriosis^a^ | Adenovirus (Group F serotypes 40 & 41) | 169 | 181 | 198 | 152 | 135 | 45 | 47 |  |
|  | Astrovirus | 66 | 49 | 78 | 397 | 291 | 284 | 304 |  |
|  | *Campylobacter* | 57,926 | 59,262 | 60,943 | 57,046 | 58,156 | 55,643 | 50,348 |  |
|  | Cryptosporidium | 4,471 | 3,490 | 3,199 | 5,331 | 3,129 | 3,579 | 5,354 |  |
|  | STEC (shiga toxin-producing *E. coli*) | 933 | 1,003 | 871 | 725 | 784 | 780 | 430^f^ |  |
|  | Giardia | 3,398 | 3,676 | 3,640 | 3,594 | 3,513 | 3,972 | 4,398 |  |
|  | Listeria | 179 | 167 | 159 | 178 | 182 | 162 | 188 |  |
|  | Norovirus | 12,216 | 7,784 | 8,669 | 9,459 | 4,761 | 7,635 | 6,313 |  |
|  | Rotavirus | 15,245 | 14,894 | 14,935 | 14,686 | 4,429 | 4,430 | 2,345 |  |
|  | Salmonella (excl. typhi & paratyphi) | 8,824 | 8,306 | 7,300 | 7,088 | 6,538 | 7,888 | 7,946 |  |
|  | Shigella | 1,554 | 1,932 | 1,727 | 1,935 | 2,029 | 2,184 | 1,848 |  |
|  | Total | 104,981 | 100,744 | 101,719 | 100,591 | 83,947 | 86,602 | 79,521 |  |
| HES^b^ | Primary diagnoses | 227,000 | 220,200 | 233,100 | 247,400 | 226,300 | 236,600 | 230,800 |  |
|  | Primary diagnosis (bed-days) | 568,400 | 507,400 | 495,100 | 507,500 | 459,900 | 471,500 | 434,100 |  |
|  | Secondary diagnoses | 220,900 | 219,300 | 228,700 | 243,100 | 234,900 | 258,500 | 267,000 |  |
|  | ~ of which day cases | 24,300 | 26,900 | 30,600 | 30,800 | 31,500 | 35,400 | 39,100 |  |
|  | All diagnoses | 448,000 | 439,500 | 461,800 | 490,400 | 461,200 | 495,100 | 497,800 |  |
| HNORS^c^ | Outbreaks | 1,900 | 1,200 | 1,600 | 1,500 | 600 | 900 | 500 |  |
|  | Patients | 19,500 | 11,500 | 15,500 | 14,000 | 5,400 | 7,700 | 4,300 |  |
|  | staff absences | 5,200 | 3,000 | 3,700 | 3,500 | 1,400 | 2,100 | 1,300 |  |
|  | lost bed-days | 22,900 | 15,300 | 17,200 | 16,900 | 7,400 | 12,400 | 7,200 |  |
| NHSE^d^ | unoccupied bed-days | n/a^e^ | 24,100-31,700 | 32,100-42,600 | 32,600-41,900 | 19,800-25,500 | 24,800-32,700 | 8,700-11,500 |  |
| Abbreviations: HES: hospital episode statistics, HNORS: Hospital Norovirus Outbreak Reporting System, NESSS: National Enhanced Surveillance System for STEC, NHSE: National Health Service England, SGSS: Second Generation Surveillance System, STEC: shiga toxin-producing *E. coli*.  a: Note that the aggregate figures do not account for seasonality within each year.  b: Figures of finished consultant episodes were extracted for financial years but are shown here per season, i.e. week 27 in the first year to week 26 in the following year.  c: Recording started in week 1 in 2009; values presented here represent seasons running from week 27 in the first year to week 26 in the second year; thus e.g. the first season 2009/10 covered the period of week 27 in 2009 to week 26 in 2010 [3].  d: Missing values on weekends and public holidays were imputed in best-to-worst-case scenarios; values presented here were recorded between November and March for 16, 18, 17, 21, 21, and 13 weeks for the six seasons, respectively; for a sound comparison see [4].  e: The first winter recorded by NHS England was in 2010/11.  f: For season 2015/16, no reports for shiga toxin-producing *E. coli* (STEC) were available for the year 2016 due to the data being audited. | | | | | | | | | |


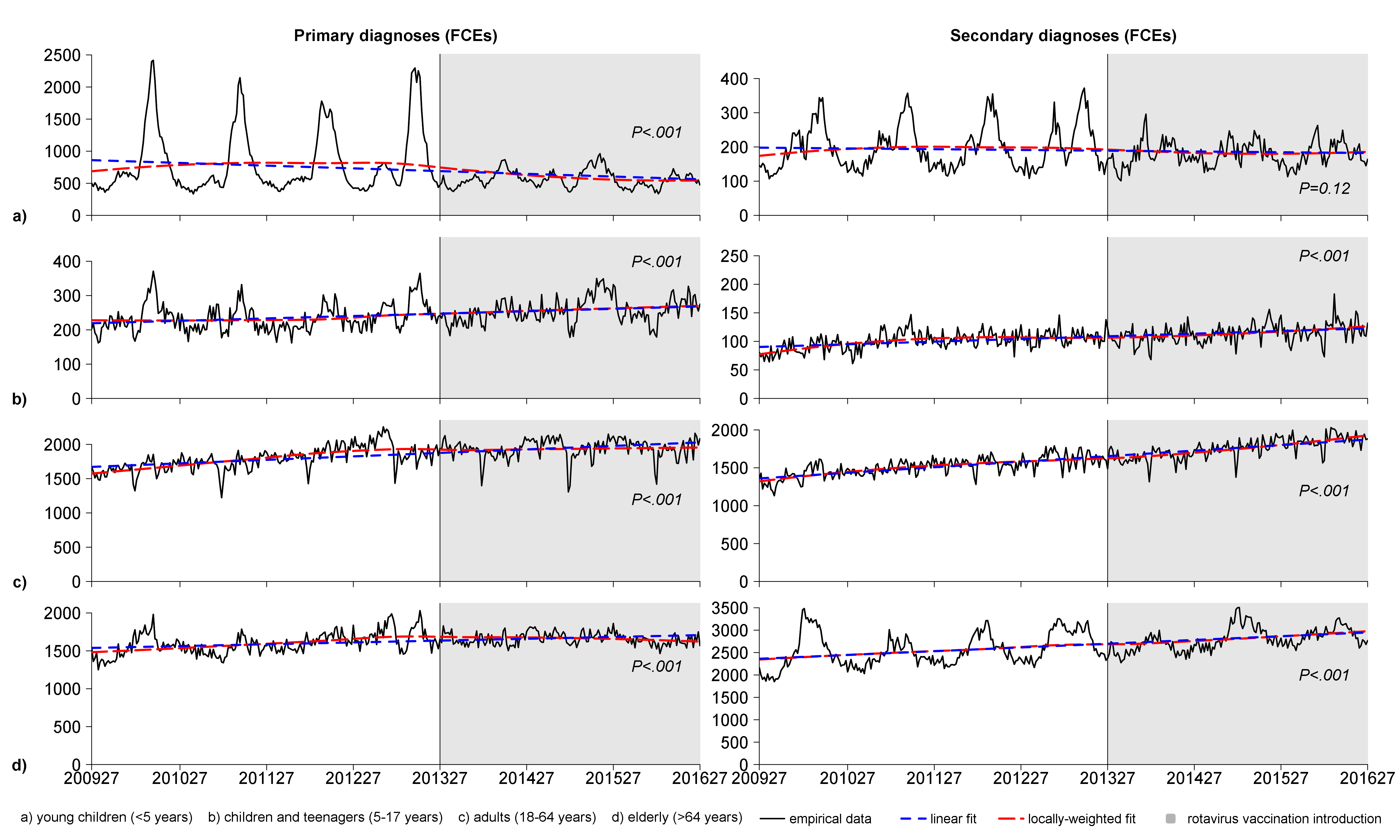
Figure 3: Additive decomposition of national hospital statistics into age-stratified inpatients with primary and secondary gastrointestinal diagnoses in England, July 2009 to June 2016 (cave: different scale of y-axes).

#### Laboratory data of gastrointestinal pathogens

We obtained the weekly number of de-duplicated laboratory reports using the date of the first specimen from faeces and lower gastrointestinal tract from reports submitted by microbiology laboratories across England to Public Health England. While reporting to Public Health England is mandatory for some enteric pathogens like *Salmonella*, it is voluntary for e.g. norovirus and rotavirus; however, laboratory testing and reporting practices have been confirmed to be high and consistent in a survey for rotavirus before [5].

For rotavirus, laboratory reports showed a decrease after July 2013 when the vaccine was introduced [2], while for norovirus they remained relatively constant with peaks in 2009/10 and 2012/13 that corresponded to novel strain emergences [6] (Supplementary Table 3). At the same time, the total number of laboratory reports decreased after July 2013 from a median 101,200 (IQR: 100,700–102,500) reports before mid-2013 to 83,900 (IQR: 81,700–85,300) afterwards (Supplementary Table 3). Note: The aggregate figures of pathogens do not account for seasonality within the years, which is why the regression models based on the weekly data provide more accurate results than a comparison of the raw data.

#### Patient-level data of norovirus infections from a local hospital

This study used individual-level patient data collected in a teaching hospital in London during a norovirus outbreak in 2015. A research nurse visited affected wards daily to collect information on new cases, bed closures and to map the movement of patients between wards. Additionally, the routinely collected data on the infection status with norovirus genogroup II (GII) were captured for an additional two weeks before and after the outbreak, i.e. 43 days in total each year.

During the outbreak in 2015 the lower daily admission than discharge rates (of 0.208 vs. 0.212, respectively) corresponded to the outbreak potentially having prevented new admissions. At the time of the outbreak, the four wards had a capacity of 56, 59, 56 and 43 beds, which is larger than the median 20 beds (range 1‒38) reported previously for 171 units in another region in England in 2002‒2003 [7]; however, the wards included one admission ward, one general ward and two infectious disease wards with isolation bed capacity. As such, we assumed that the setting was negligible for estimating the excess length of stay of patients with norovirus (it may rather have had a positive effect on the speed of containing the outbreak).

For our analysis of the routinely collected patient data, we copied for duplicate records of the same patients any missing personal information (i.e., date of birth and sex) and any missing information of the same stay when a transfer occurred merely between specialties, which led to another record being created (i.e., admission and discharge). For all records, we approximated missing information on the length of hospital stay with the stay on the wards.

We removed duplicate records of the same stay but not for different stays. By using the first positive norovirus GII infection sample during the hospital stay we ensured norovirus patients were not double counted as we excluded records of re-admissions when no other norovirus GII infection sample was taken. For stays beyond our observation period, the infection status was unknown and we censored records to these time points.

In order to obtain unbiased controls without gastroenteritis, patients were excluded from the analysis that had a) any secondary infectious or non-infectious intestinal disease codes (A00‒A09; K528 and K529), b) a primary non-infectious intestinal disease code (K528 and K529) or c) a negative PCR test taken for norovirus GII due to potentially being infected with a different norovirus strain, or a different enteric pathogen (e.g. 43.5% of cases during the local outbreak in 2015 were symptomatic yet tested norovirus GII negative, while 17.4% of cases were positively tested but asymptomatically infected). For the demographic characteristics of all patients, together with those excluded, see Supplementary Table 4.

| Table 4: Demographic characteristics of the local sample of patients from a teaching hospital in London, England, on the wards affected by the norovirus outbreak of May 31 to June 15, 2015, and the previous two years. | | | | | | | | |
| --- | --- | --- | --- | --- | --- | --- | --- | --- |
| Variables | **All patients** |  | **Excluded patients** |  | **All patients analysed** |  | **All controls** | **All cases** |
| Patients, n (%) | 2,855 (100.0%) |  | 346 (12.0%) |  | 2,509 (88.0%) |  | 2,465 (86.3%) | 44 (1.5%) |
| Age (years), mean (SD) | 60.1 (20.4) |  | 66.5 (21.0) |  | 59.2 (20.2) |  | 59.1 (20.1) | 67.1 (20.9) |
| Sex (female),  n (%) | 1,456 (51.0%) |  | 191 (55.2%) |  | 1,265 (50.4%) |  | 1,237 (50.2%) | 28 (63.6%) |
| CCI score (>0), n (%) | 1,667 (58.4%) |  | 227 (65.6%) |  | 1,440 (57.4%) |  | 1,408 (57.1%) | 32 (72.7%) |
| In-hospital mortality, n (%) | 77 (2.7%) |  | 23 (6.6%) |  | 54 (2.2%) |  | * | * |
| LOS (days), mean (range) | 6.3 (0, 43) |  | 15.4 (0, 43) |  | 5.0 (0, 43) |  | 4.9 (0, 43) | 12.6 (0, 43) |
| Excess LOS (days), mean (95% CI)^a^ | n/a |  | n/a |  | n/a |  | n/a | n/a |
| QALY gain (undiscounted), mean (95% CI)^b^ | 0.183  (0.0001, 0.386) |  | 0.220  (0.0002, 0.397) |  | 0.179  (0.0001, 0.386) |  | 0.178  (0.0001, 0.386) | 0.193  (0.0004, 0.349) |
| QALY gain (discounted), mean (95% CI)^b^ | 0.147  (0.0001, 0.298) |  | 0.182  (0.0002, 0.312) |  | 0.142  (0.0001, 0.293) |  | 0.142  (0.0001, 0.293) | 0.160  (0.0004, 0.288) |
| CCI: Charlson comorbidity index, CI: confidence interval, GII: norovirus genogroup II, LOS: length of stay, n/a: not applicable, PCR: polymerase chain reaction, QALY: quality-adjusted life year, SD: standard deviation.  a: No excess LOS is presented here given that hospitalisations for a primary infectious intestinal disease but without laboratory-confirmed norovirus diagnosis cannot necessarily be categorised as an excess stay.  b: For cases, the QALYs gained were driven by the high level of comorbidities. If we approximate the gastroenteritis-related health gain by subtracting the QALY gain of all non-gastroenteritis control patients from the QALY gain of all gastroenteritis cases, we derive 0.160-0.142=0.018 QALYs gained.  ‘*’ in this table means a figure between 1 and 5, values suppressed to prevent possible identification of individuals [1]. | | | | | | | | |

1. Details on the linear regression analysis

In order to attribute norovirus to inpatients with gastroenteritis, we estimated the expected number of gastroenteritis cases caused by different gastrointestinal pathogens per week using multiple linear regressions with laboratory reports for the pathogens as explanatory variables. Our linear regression estimated the expected number of gastroenteritis inpatients *Y* using the gastrointestinal pathogens in week *j*:

$$Y_{j}=c+\sum\alpha_{i}L_{ij}$$

where *L_ij_* denotes the number of laboratory reports for the gastrointestinal pathogens *i* in week *j*, α*_i_* is the regression coefficient for pathogen *i* to estimate the number of inpatients with gastroenteritis diagnoses associated with each laboratory report, and *c* is a constant term for the background number of gastrointestinal illnesses that the model was not able to attribute to the weekly observations of laboratory reports. Similar to previous studies [8-10], the initial model included all pathogens, which were subsequently removed stepwise backwards when they were not significantly contributing to the model (i.e., *P*<.05 and their removal did not decrease the adjusted R^2^), or that had a biologically implausible negative coefficient. We then multiplied the regression coefficient per pathogen with its observed number of laboratory reports before deriving the attributed fractions from the fitted total of hospital cases. We performed various sensitivity analyses of excluding the constant, using only norovirus as explanatory variable, and separating the data to account for rotavirus vaccine introduction in July 2013. In a separate analysis, we limited the data up to December 2015 to be able to include shiga toxin-producing *E. coli* (STEC).

The regression models with the highest adjusted R^2^ were those that separated the dataset in mid-2013 to account for rotavirus vaccination introduction, excluded the constant, and controlled for other significant pathogens besides norovirus, particularly astrovirus, *Campylobacter*, *Giardia*, *Listeria*, rotavirus and *Shigella* (Supplementary Table 5). Our best-fitting models confirmed previous studies on rotavirus vaccination [11, 12] by showing a significant decrease in the burden attributable to rotavirus admissions following vaccine introduction in July 2013.

The results of the separate analysis that limited the data to December 2015 to include STEC showed slightly lower results for norovirus that were not significant when separating the dataset in mid-2013 for rotavirus vaccination introduction (Supplementary Table 6). Because STEC was excluded in all but one regression model (and not the best-fitting one), where it accounted for only 1.6% (CI: 0.5%–2.4%) of primary diagnoses, we proceeded without STEC in the interest of being able to use a longer dataset that captured the full seasonal activity of norovirus in 2015/16 [3].

We also checked how the regression models would predict the norovirus-attributable burden for each season and visualised results for the best-fitting models (Supplementary Figure 4). Despite a decreased power from fewer observations, proportions of above 20% were found for norovirus-attributable primary gastroenteritis for each season after mid-2013 (despite not including a novel strain emergence). The increase to about 25% already in 2012/13 corresponded with the Sydney/2012 norovirus strain emergence [6], while the consistently high levels afterwards corresponded with the rotavirus vaccination introduction [2]. For secondary diagnoses, the fluctuations also correspond to the novel strain emergences in 2009/10 and 2012/13 (where values are at about 30%, while most other seasons are at lower levels of 20%) and the fewer reported norovirus outbreaks in HNORS in recent seasons. The strain emergence in 2012 was also much more efficient in transmission than the one in 2009, which may explain the lower fraction of primary diagnoses but higher fraction of secondary diagnoses in 2009/2010 (Supplementary Figure 4).


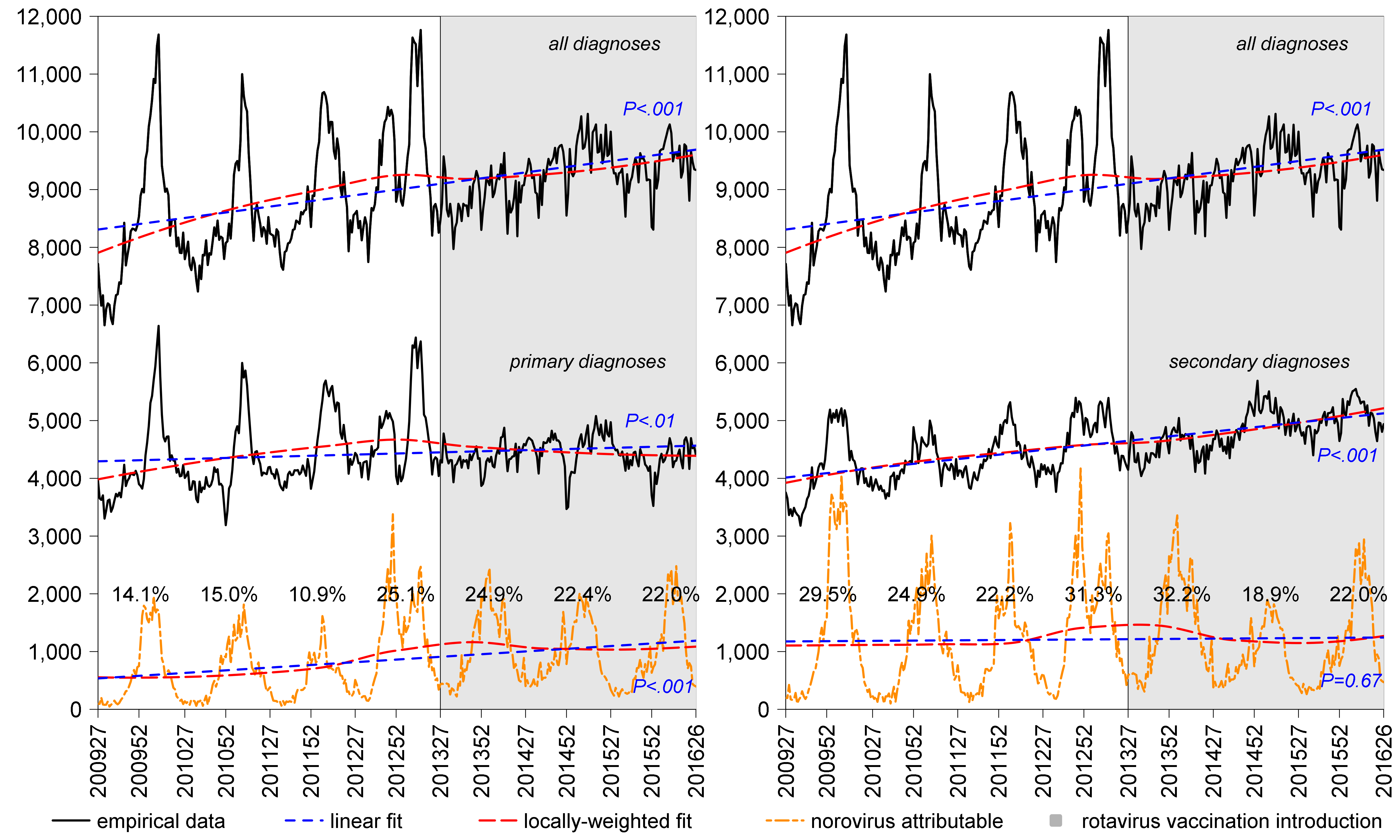
Figure 4: National hospital statistics for inpatients with infectious and non-infectious gastrointestinal illnesses in England, July 2009 to June 2016, and visualising norovirus-associated gastroenteritis using linear regressions fitted to the data per season.

Of note, a previous regression analysis found no increase in the number of (voluntarily-reported) norovirus outbreaks when using positive norovirus laboratory cases as explanatory variable [13]. Differences to our findings here may be explained by the fact that we included a wider range of enteric pathogens as explanatory variables and we used as response variable the obligatory hospital diagnosis codes recorded for each gastrointestinal hospitalisation episode nationally.

| Table 5: Attributable cause of gastrointestinal diagnoses by regression model, 2009/2010 to 2015/2016 | | | | | | | | | | | | | | |  |
| --- | --- | --- | --- | --- | --- | --- | --- | --- | --- | --- | --- | --- | --- | --- | --- |
| **Data, timeframe, model** | | **Adenovirus** | **Astrovirus** | ***Campylobacter*** | **Cryptospo-ridium** | **Giardia** | **Listeria** | **Norovirus** | **Rotavirus** | **Salmonella (excl. typhi & paratyphi)** | **Shigella** | **Intercept** | **Adj. R^2^** | | |
| **Primary gastrointestinal diagnoses (FCEs)** | | | | | | | | | | | | | |  |  |
| 2009/10- 2015/16 | Base analysis | - | 3.3 (3.3–3.3) | 8.8 (7.0–10.1) | 0.7 (-0.6–1.6) | 5.3 (2.3–7.4) | - | 2.5 (1.7–3.1) | 6.0 (5.6–6.6) | - | 4.0 (1.9–5.5) | 69.3 (63.3–77.9) | 0.761 | | |
|  | No constant | **-** | **5.5** (5.5–5.5) | **35.8** (33.6–38.8) | - | **23.8** (23.5–24.0) | **4.8** (3.6–5.6) | **11.7** (11.3–12.3) | **6.5** (6.3–6.9) | - | **11.9** (9.4–13.6) | - | **0.989** | | |
|  | Noro only | - | - | - | - | - | - | 7.8 (6.8–8.8) | - | - | - | 92.2 (91.2–93.2) | 0.295 | | |
| 2009/10- 2012/13 | Base analysis | - | 2.6 (2.5–2.7) | 12.7 (10.8–14.1) | 2.3 (1.4–3.0) | - | - | 3.7 (3.0–4.3) | 9.4 (8.9–10.1) | - | 1.7 (-0.9–3.7) | 67.5 (63.3–73.1) | 0.903 | | |
|  | No constant | **-** | **3.3** (3.1–3.4) | **39.3** (36.2–44.2) | - | **20.3** (18.5–21.5) | **4.7** (3.2–5.6) | **12.4** (11.8–13.3) | **10.8** (10.0–12.2) | - | **9.2** (5.5–11.6) | - | **0.991** | | |
|  | Noro only | - | - | - | - | - | - | 11.1 (9.7–12.3) | - | - | - | 88.9 (87.7–90.3) | 0.393 | | |
| 2013/14- 2015/16 | Base analysis | - | - | 12.8 (11.4–13.9) | - | 5.7 (3.1–7.5) | - | 5.8 (5.1–6.2) | 4.3 (4.1–4.5) | 3.6 (1.5–5.1) | - | 67.8 (62.7–74.7) | 0.578 | | |
|  | No constant | **-** | **3.2** (1.7–3.9) | **42.5** (36.9–53.2) | **4.4** (1.7–5.8) | **15.1** (10.7–17.4) | **4.5** (3.3–5.1) | **17.7** (15.6–21.6) | **6.3** (6.2–6.5) | - | **6.4** (1.3–9.0) | - | **0.993** | | |
|  | Noro only | - | - | - | - | - | - | 0.8 (-0.8–2.3) | - | - | - | 99.2 (97.7–100.8) | 0.000 | | |
| **Cases with secondary gastrointestinal diagnoses (FCEs)** | | | | | | | | | | | | | | |  |
| 2009/10- 2015/16 | Base analysis | - | 4.0 (3.7–4.3) | - | - | 6.6 (3.4–9.3) | - | 6.0 (5.5–6.4) | - | - | - | 83.3 (80.0–87.4) | 0.482 | | |
|  | No constant | **-** | **6.3** (6.2–6.4) | **27.8** (27.0–29.1) | **-** | **30.2** (29.6–31.1) | **5.0** (3.4–6.1) | **17.8** (16.1–20.5) | **-** | **-** | **12.9** (9.5–15.1) | - | **0.980** | | |
|  | Noro only | - | - | - | - | - | - | 6.7 (5.8–7.6) | - | - | - | 93.3 (92.4–94.2) | 0.273 | | |
| 2009/10- 2012/13 | Base analysis | - | 2.3 (2.0–2.5) | - | - | - | - | 8.6 (8.2–9.0) | 0.7 (-0.1–1.3) | - | 2.4 (-0.4–4.8) | 86.0 (82.4–90.3) | 0.750 | | |
|  | No constant | - | **2.7** (2.3–3.0) | **34.4** (31.8–39.4) | - | **22.0** (20.0–23.0) | **5.0** (3.0–6.0) | **20.2** (17.7–24.7) | **2.7** (1.5–3.4) | - | **13.0** (9.2–15.0) | - | **0.984** | | |
|  | Noro only | - | - | - | - | - | - | 10.3 (9.5–11.0) | - | - | - | 89.7 (89.0–90.5) | 0.641 | | |
| 2013/14- 2015/16 | Base analysis | - | 0.8 (-0.5–1.8) | - | - | 6.6 (3.4–9.1) | - | 7.9 (7.1–8.5) | - | - | - | 84.8 (80.6–90.0) | 0.484 | | |
|  | No constant | - | **4.7** (3.2–5.5) | **40.2** (35.4–49.9) | **5.1** (1.2–7.1) | **20.0** (14.6–22.7) | **4.5** (2.2–5.6) | **23.8** (20.6–29.9) | **1.8** (-0.9–3.1) | - | - | - | **0.988** | | |
|  | Noro only | - | - | - | - | - | - | 7.7 (6.5–8.7) | - | - | - | 92.3 (91.3–93.5) | 0.442 | | |
| **All gastrointestinal diagnoses** | | | | | | | | | | | | | |  |  |
| 2009/10- 2015/16 | Base analysis | - | 3.3 (3.1–3.5) | - | - | 6.0 (3.4–8.0) | - | 4.0 (3.4–4.5) | 2.8 (2.6–3.0) | - | 3.2 (0.9–5.0) | 80.7 (76.0–86.6) | 0.635 | | |
|  | No constant | **-** | **5.9** (5.8–6.0) | **31.8** (30.1–34.3) | - | **26.9** (26.7–27.4) | **4.9** (3.6–5.8) | **14.8** (13.9–16.2) | **3.4** (3.0–3.6) | - | **12.3** (9.6–14.2) | - | **0.986** | | |
|  | Noro only | - | - | - | - | - | - | 7.3(6.6–8.0) | **-** | - | - | 92.7(92.0–93.4) | 0.388 | | |
| 2009/10- 2012/13 | Base analysis | - | 2.5 (2.4–2.6) | 5.0 (2.1–7.3) | - | - | - | 5.8 (5.3–6.1) | 5.1 (5.1–5.1) | - | 2.7 (0.5–4.4) | 78.9 (74.5–84.6) | 0.868 | | |
|  | No constant | **-** | **3.0** (2.7–3.1) | **36.8** (34.0–41.6) | - | **21.1** (19.3–22.2) | **4.8** (3.2–5.8) | **16.2** (14.8–18.5) | **7.0** (6.7–7.3) | - | **11.0** (7.3–13.2) | - | **0.989** | | |
|  | Noro only | - | - | - | - | - | - | 10.7 (9.8–11.5) | - | - | - | 89.3 (88.5–90.2) | 0.576 | | |
| 2013/14- 2015/16 | Base analysis | - | **-** | 6.5 (4.0–8.4) | - | 6.4 (4.4–8.0) | - | 6.8 (6.3–7.2) | 1.3 (0.6–1.8) | - | - | 79.0 (74.6–84.8) | 0.467 | | |
|  | No constant | **-** | **4.1** (2.6–4.9) | **42.9** (38.1–51.5) | **5.1** (2.0–6.8) | **18.4** (13.6–21.2) | **4.4** (2.6–5.4) | **21.1** (18.9–25.0) | **4.0** (2.7–4.7) | - | - | - | **0.991** | | |
|  | Noro only | - | - | - | - | - | - | 4.4 (3.4–5.3) | - | - | - | 95.6 (94.7–96.6) | 0.299 | | |
| FCE: Finished Consultant Episode, NHS: National Health Service.  The results of the most parsimonious models with the highest goodness-of-fit are presented in bold. 95% confidence intervals are given in parentheses. | | | | | | | | | | | | | | |  |

| Table 6: Attributable cause of gastrointestinal diagnoses by regression model, 2009/2010 to December 2015 in order to be able to include STEC | | | | | | | | | | | | | | |
| --- | --- | --- | --- | --- | --- | --- | --- | --- | --- | --- | --- | --- | --- | --- |
| **Data, timeframe, model** | | **Adeno-virus** | **Astrovirus** | ***Campylobacter*** | **Cryptospo-ridium** | ***STEC* (shiga toxin-producing *E. coli*)** | **Giardia** | **Listeria** | **Norovirus** | **Rotavirus** | **Salmonella (excl. typhi  & paratyphi)** | **Shigella** | **Intercept** | **Adj. R^2^** |
| **Primary gastrointestinal diagnoses (FCEs)** | | | | | | | | | | | | | | |
| 2009/10- Dec 2015 | Base analysis | - | 3.1 (3.0–3.1) | 9.7 (7.8–11.1) | - | - | 6.1 (3.7–7.9) | - | 2.3 (1.4–2.9) | 6.6 (6.2–7.1) | - | 5.0 (2.8–6.5) | 67.2 (62.2–74.2) | 0.774 |
|  | No constant | - | **4.9** (4.9–5.0) | **37.2** (34.8–40.6) | - | - | **22.6** (21.9–23.0) | **4.8** (3.5–5.6) | **10.6** (10.3–10.9) | **7.4** (7.1–7.9) | - | **12.6** (10.2–14.2) | - | **0.989** |
|  | Noro only | - | - | - | - | - | - | - | 8.0 (6.9–8.9) | - | - | - | 92.0 (91.1–93.1) | 0.307 |
| 2009/10- 2012/13 | Base analysis | - | 2.6 (2.5–2.7) | 12.7 (10.8–14.1) | 2.3 (1.4–3.0) | - | - | - | 3.7 (3.0–4.3) | 9.4 (8.9–10.1) | - | 1.7 (-0.9–3.7) | 67.5 (63.3–73.1) | 0.903 |
|  | No constant | - | **3.3** (3.1–3.4) | **39.3** (36.2–44.2) | - | - | **20.3** (18.5–21.5) | **4.7** (3.2–5.6) | **12.4** (11.8–13.3) | **10.8** (10.0–12.2) | - | **9.2** (5.5–11.6) | - | **0.991** |
|  | Noro only | - | - | - | - | - | - | - | 11.1 (9.7–12.3) | - | - | - | 88.9 (87.7–90.3) | 0.393 |
| 2013/14- Dec 2015 | Base analysis | - | 1.0 (-0.02–1.7) | 13.2 (11.8–14.0) | - | 1.6 (0.5–2.4) | 6.4 (3.9–8.1) | - | 5.1 (4.4–5.6) | 5.1 (5.1–5.2) | 3.7 (1.1–5.3) | - | 63.9 (57.8–73.1) | 0.625 |
|  | No constant | - | **3.4** (2.1–4.0) | **43.3** (37.2–55.3) | - | - | **16.3** (12.4–18.3) | **4.0** (2.4–4.9) | **13.8** (12.7–16.2) | **7.6** (7.2–8.4) | - | **7.1** (1.6–10.0) | - | **0.994** |
|  | Noro only | - | - | - | - | - | - | - | 1.2 (-0.6–2.8) | - | - | - | 98.8 (97.2–100.6) | 0.005 |
| **Cases with secondary gastrointestinal diagnoses (FCEs)** | | | | | | | | | | | | | | |
| 2009/10- Dec 2015 | Base analysis | - | 3.4 (3.2–3.6) | - | - | - | 5.7 (2.3–8.4) | - | 6.3 (6.0–6.5) | - | - | 3.7 (0.6–6.1) | 80.9 (75.4–87.9) | 0.492 |
|  | No constant | - | **5.3** (5.3–5.3) | **29.7** (28.6–31.6) | - | - | **28.2** (28.0–28.5) | **4.7** (2.9–5.8) | **17.5** (15.7–20.2) | **-** | - | **14.6** (11.6–16.5) | - | **0.981** |
|  | Noro only | - | - | - | - | - | - | - | 6.5 (5.6–7.4) | - | - | - | 93.5 (92.6–94.4) | 0.289 |
| 2009/10- 2012/13 | Base analysis | - | 2.3 (2.0–2.5) | - | - | - | - | - | 8.6 (8.2–9.0) | 0.7 (-0.1–1.3) | - | 2.4 (-0.4–4.8) | 86.0 (82.4–90.3) | 0.75 |
|  | No constant | - | **2.7** (2.3–3.0) | **34.4** (31.8–39.4) | - | - | **22.0** (20.0–23.0) | **5.0** (3.0–6.0) | **20.2** (17.7–24.7) | **2.7** (1.5–3.4) | - | **13.0** (9.2–15.0) | - | **0.984** |
|  | Noro only | - | - | - | - | - | - | - | 10.3 (9.5–11.0) | - | - | - | 89.7 (89.0–90.5) | 0.641 |
| 2013/14- Dec 2015 | Base analysis | - | - | - | - | - | 8.6 (5.4–11.2) | - | 7.8 (7.1–8.5) | - | - | - | 83.6 (80.3–87.5) | 0.465 |
|  | No constant | - | **4.4** (2.8–5.1) | **41.2** (36.0–51.9) | **5.7** (1.9–7.6) | - | **21.9** (17.6–24.0) | **3.3** (0.03–4.9) | **20.3** (18.1–24.6) | **3.2** (1.0–4.3) | - | - | - | **0.989** |
|  | Noro only | - | - | - | - | - | - | - | 6.8 (5.6–8.0) | - | - | - | 93.2 (92.0–94.4) | 0.382 |
| **All gastrointestinal diagnoses** | | | | | | | | | | | | | | |
| 2009/10- Dec 2015 | Base analysis | - | 3 (2.7–3.1) | - | - | - | 5.9 (3.2–8.0) | - | 3.8 (3.1–4.3) | 3.3 (3.1–3.4) | - | 4.8 (2.5–6.5) | 79.4 (74.7–85.3) | 0.66 |
|  | No constant | - | **5.2** (5.1–5.2) | **33.3** (31.4–36.2) | - | - | **25.4** (25.4–25.5) | **4.7** (3.3–5.7) | **13.6** (12.8–14.7) | **4.2** (4.0–4.4) | - | **13.6** (11.0–15.2) | **-** | **0.987** |
|  | Noro only | - | - | - | - | - | - | - | 7.3 (6.5–8.0) | - | - | - | 92.7 (92.0–93.5) | 0.394 |
| 2009/10- 2012/13 | Base analysis | - | 2.5 (2.4–2.6) | 5 (2.1–7.3) | - | - | - | - | 5.8 (5.3–6.1) | 5.1 (5.1–5.1) | - | 2.7 (0.5–4.4) | 78.9 (74.5–84.6) | 0.868 |
|  | No constant | - | **3** (2.7–3.1) | **36.8** (34.0–41.6) | - | - | **21.1** (19.3–22.2) | **4.8** (3.2–5.8) | **16.2** (14.8–18.5) | **7** (6.7–7.3) | - | **11** (7.3–13.2) | **-** | **0.989** |
|  | Noro only | - | - | - | - | - | - | - | 10.7 (9.8–11.5) | - | - | - | 89.3 (88.5–90.2) | 0.576 |
| 2013/14- Dec 2015 | Base analysis | - | 0.6 (-0.4–1.2) | 9.4 (7.0–11.0) | 1.3 (-0.3–2.5) | - | 6.4 (3.3–8.6) | - | 6.5 (6.1–6.8) | 2 (1.4–2.5) | - | - | 73.8 (67.5–82.8) | 0.509 |
|  | No constant | - | **4** (2.6–4.8) | **44** (38.8–53.4) | **5.3** (2.1–7.0) | - | **20.4** (16.4–22.6) | **3.6** (1.2–4.9) | **17.5** (16.1–20.0) | **5.2** (4.4–5.7) | - | - | - | **0.992** |
|  | Noro only | - | - | - | - | - | - | - | 4.1 (3.0–5.2) | - | - | - | 95.9 (94.8–97.0) | 0.255 |
| FCE: Finished Consultant Episode, NHS: National Health Service, STEC: shiga toxin-producing *E. coli*.  The results of the most parsimonious models with the highest goodness-of-fit are presented in bold. 95% confidence intervals are given in parentheses. | | | | | | | | | | | | | | |


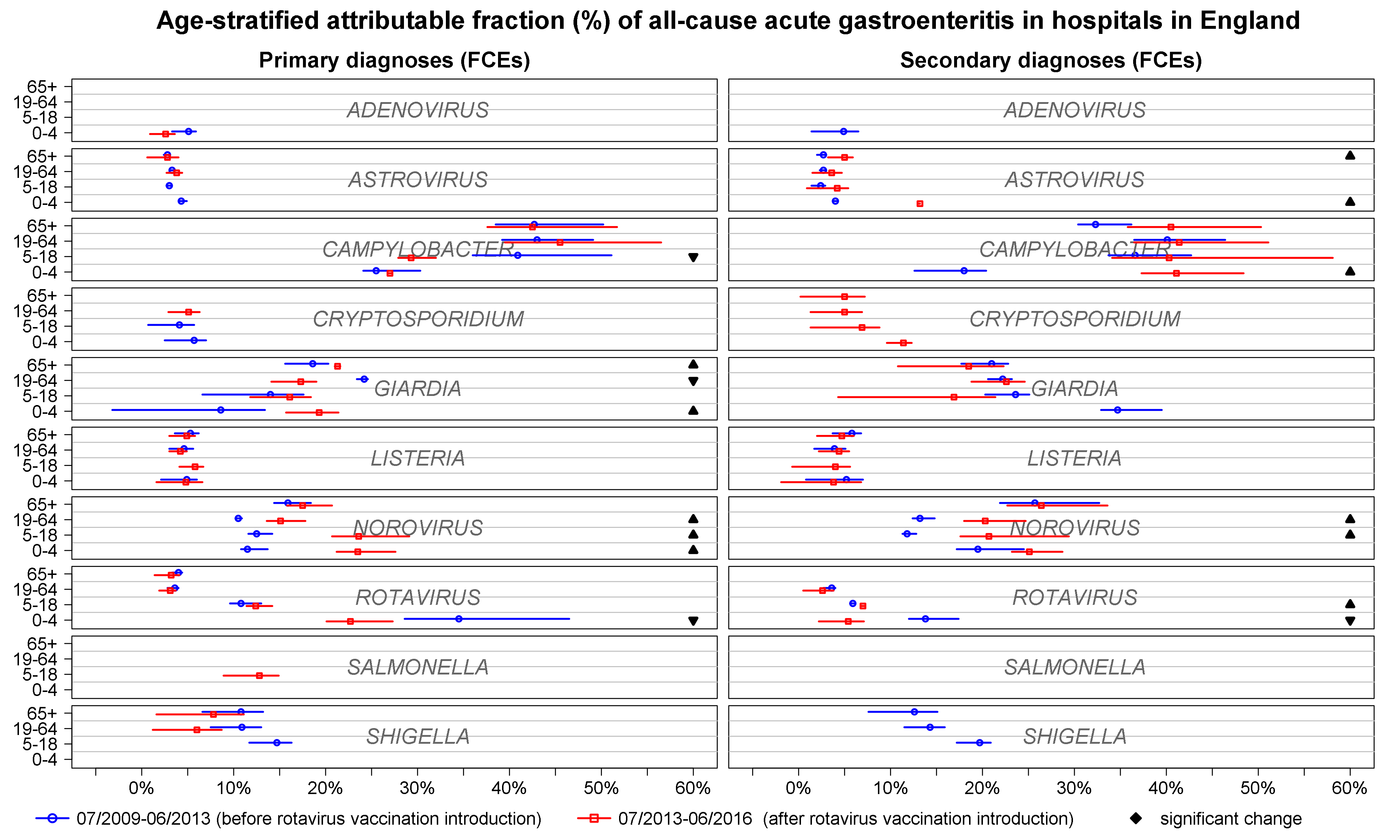
Figure 5: Age-stratified attributable fraction (in %) of enteric pathogens on all-cause acute gastrointestinal primary and secondary diagnoses in hospitals in England, using linear regressions fitted to the data of July 2009 to June 2013 vs. July 2013 to June 2016.

In Figure 5, we explored the attributable fraction of enteric pathogens on gastrointestinal diagnoses stratified by age. Pathogens are presented in alphabetical order given their different impact qua proportion in different age groups. Moreover, it needs to be kept in mind that this ecological regression analysis cannot inherently explain the reasons for the changes observed (e.g. in younger ages for norovirus), although it seems likely to be impacted by reductions in attributable cases for other pathogens, particularly rotavirus. Further in-depth analyses are required that were outside of the scope of this study.

We also fitted a negative binomial regression model to the data to compare with the multivariate linear regression models used in the paper. This allowed checking for over-dispersion by the rule of thumb of the residual deviance divided by the degrees of freedom being close to 1.0. This ratio was not higher than 1.05 in any of the models, indicating no over-dispersion.

For the norovirus-attributable burden, the values are indeed close to the multivariate regression although always slightly higher (see Table 7). The rest of the conclusions are not changing either.

We then compared the Akaike information criterion (AIC) of the models, with lower values indicating a (relatively) better fitting model. Overall, the linear regression models are almost always slightly better than the negative binomial models, except for the one of cases with secondary diagnoses before July 2013. Given the small difference though and the fact that we do not use that particular model in the main results, we kept presenting our original estimates with the multivariate regression analysis.

| Table 7: Comparison of regression models; including results of norovirus-attributable proportions | | |
| --- | --- | --- |
|  | **Linear regression** | **Negative binomial regression** |
| **Primary gastrointestinal diagnoses (FCEs)** | | |
| **2009/10-2015/16** | 11.7 (11.3–12.3)  AIC: 5553 | 14.6 (13.9–15.7)  AIC: 5580  Dispersion: 1.02 |
| **2009/10-2012/13** | 12.4 (11.8–13.3)  AIC: 3132 | 15.2 (14.0-17.1)  AIC: 3141  Dispersion: 1.04 |
| **2013/14-2015/16** | 17.7 (15.6–21.6)  AIC: 2312 | 19.3 (17.5–22.3)  AIC: 2339  Dispersion: 1.05 |
| **Cases with secondary gastrointestinal diagnoses (FCEs)** | | |
| **2009/10-2015/16** | 17.8 (16.1–20.5)  AIC: 5784 | 20.0 (18.1–22.9)  AIC: 5790  Dispersion: 1.02 |
| **2009/10-2012/13** | 20.2 (17.7–24.7)  AIC: 3242 | 25.0 (21.6–31.0)  AIC: 3236  Dispersion: 1.04 |
| **2013/14-2015/16** | 23.8 (20.6–29.9)  AIC: 2432 | 26.1 (22.8–32.0)  AIC: 2453  Dispersion: 1.04 |
| **All gastrointestinal diagnoses** | | |
| **2009/10-2015/16** | 14.8 (13.9–16.2)  AIC: 6164 | 18.1 (16.8-20.2)  AIC: 6183  Dispersion: 1.02 |
| **2009/10-2012/13** | 16.2 (14.8–18.5)  AIC: 3466 | 19.7 (17.5–23.3)  AIC: 3470  Dispersion: 1.04 |
| **2013/14-2015/16** | 21.1 (18.9–25.0)  AIC: 2583 | 22.6 (20.0–27.2)  AIC: 2611  Dispersion: 1.05 |

1. Details on the multi-state model

We estimated the mean excess length of hospital stay due to norovirus using a multi-state model [14-16] consisting of four mutually exclusive states: admission, infected/diseased, discharged, and death (Supplementary Figure 6). Inpatients were allowed to make five transitions between two transient states for patients that do not, or do, develop norovirus, which are labelled in the following with “0” and “1”, respectively, and two absorbing states of being discharged alive or dead, respectively labelled with “2” and “3” (Supplementary Table 8 and Supplementary Figure 6). We decided against a combined endpoint discharged/death given the rarity of in-hospital mortality among norovirus cases in our sample, which conforms with national data [17]; for most cases, our model thus estimated the excess length of stay based on control patients discharged alive (i.e., hazard rate λ_12_ vs. λ_02_ in Supplementary Figure 6).


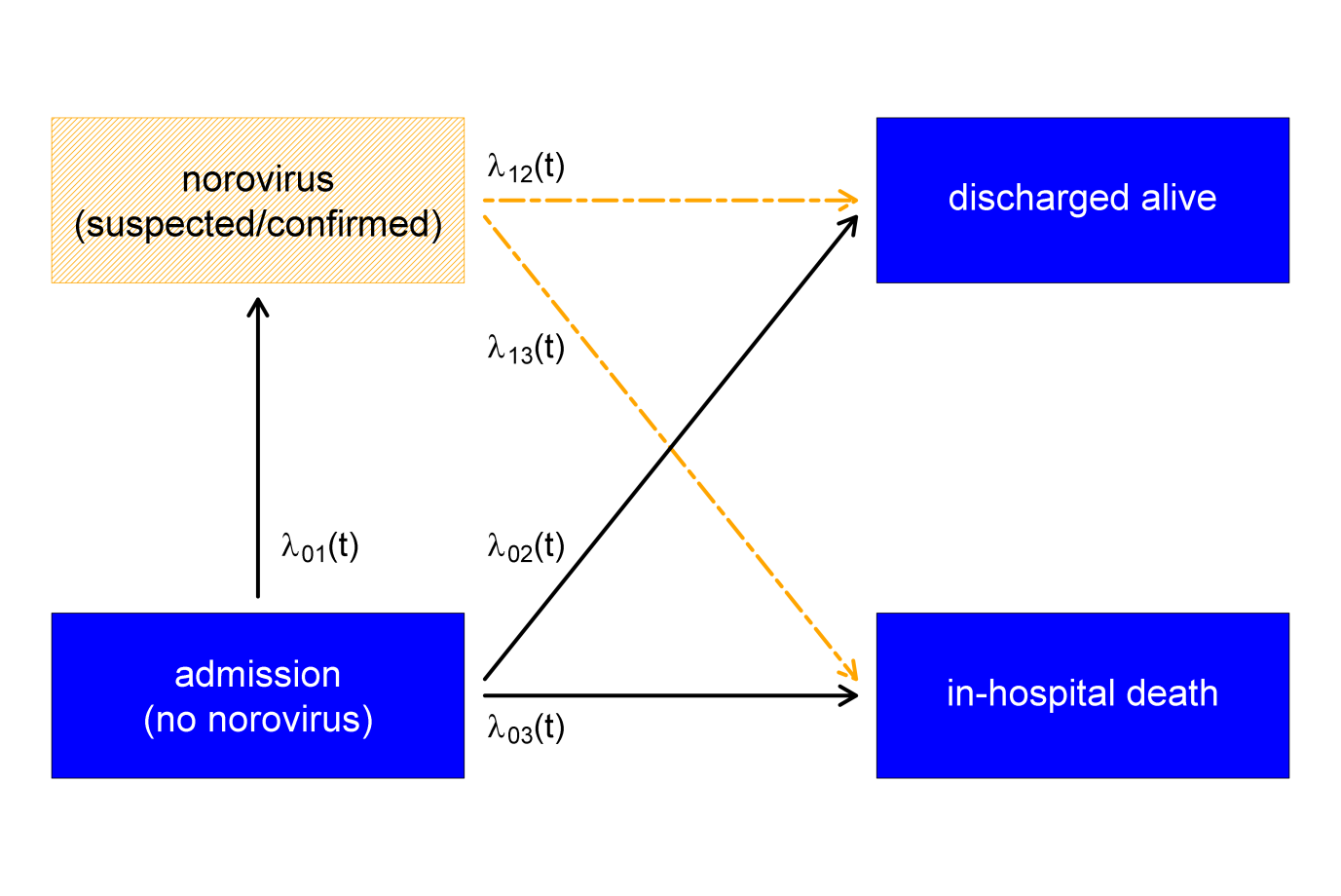
Figure 6. State-transition diagram of the multi-state model with hazard rates.

| Table 8: Possible transitions of patients in the multi-state model | | |
| --- | --- | --- |
| **Transitions** | **from** | **to** |
| Inpatients developing suspected and/or confirmed norovirus | 0 | 1 |
| Inpatients being discharged alive without developing norovirus | 0 | 2 |
| Inpatients dying in hospital without having developed norovirus | 0 | 3 |
| Inpatients being discharged alive after developing norovirus | 1 | 2 |
| Inpatients dying in hospital after having developed norovirus | 1 | 3 |

The model was populated with the local patient-level hospital data that included the time of norovirus infection and symptoms. For day cases who did not stay overnight in hospital, the resource consumption was approximated with 0.5 bed-days. For primary gastrointestinal diagnoses, we assumed that a diagnosis of primary gastrointestinal illnesses (as manifested by diarrhoea and/or vomiting) was made within about 15 minutes after being admitted (i.e., a value of 0.01).

The daily transition probabilities of patients were estimated as time-varying hazards using the empirical transition matrix (also called Aalen-Johansen estimator) [18]; cf. Supplementary Figure 7 for the 33 suspected and/or GII confirmed norovirus cases and 2,465 non-gastroenteritis controls.


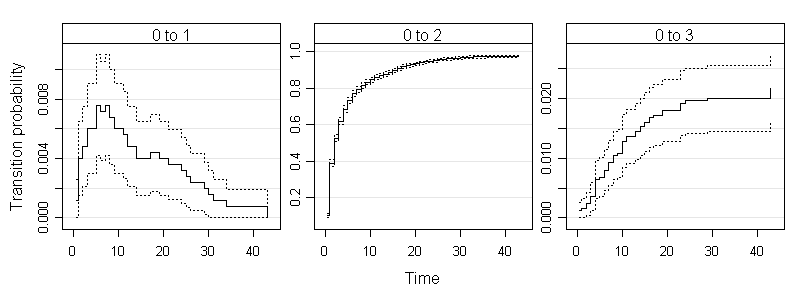
Figure 7. Transition probabilities from the individual-level patient data of developing norovirus (0 to 1), of being discharged alive without norovirus (0 to 2), of being discharged dead without norovirus (0 to 3). Probabilities for norovirus not shown here due to small numbers of in-hospital mortality [1].

Afterwards, the activity-weighted mean excess length of stay was calculated from the difference in the expected length of stay per day and the frequency of norovirus patients (Supplementary Figure 8).


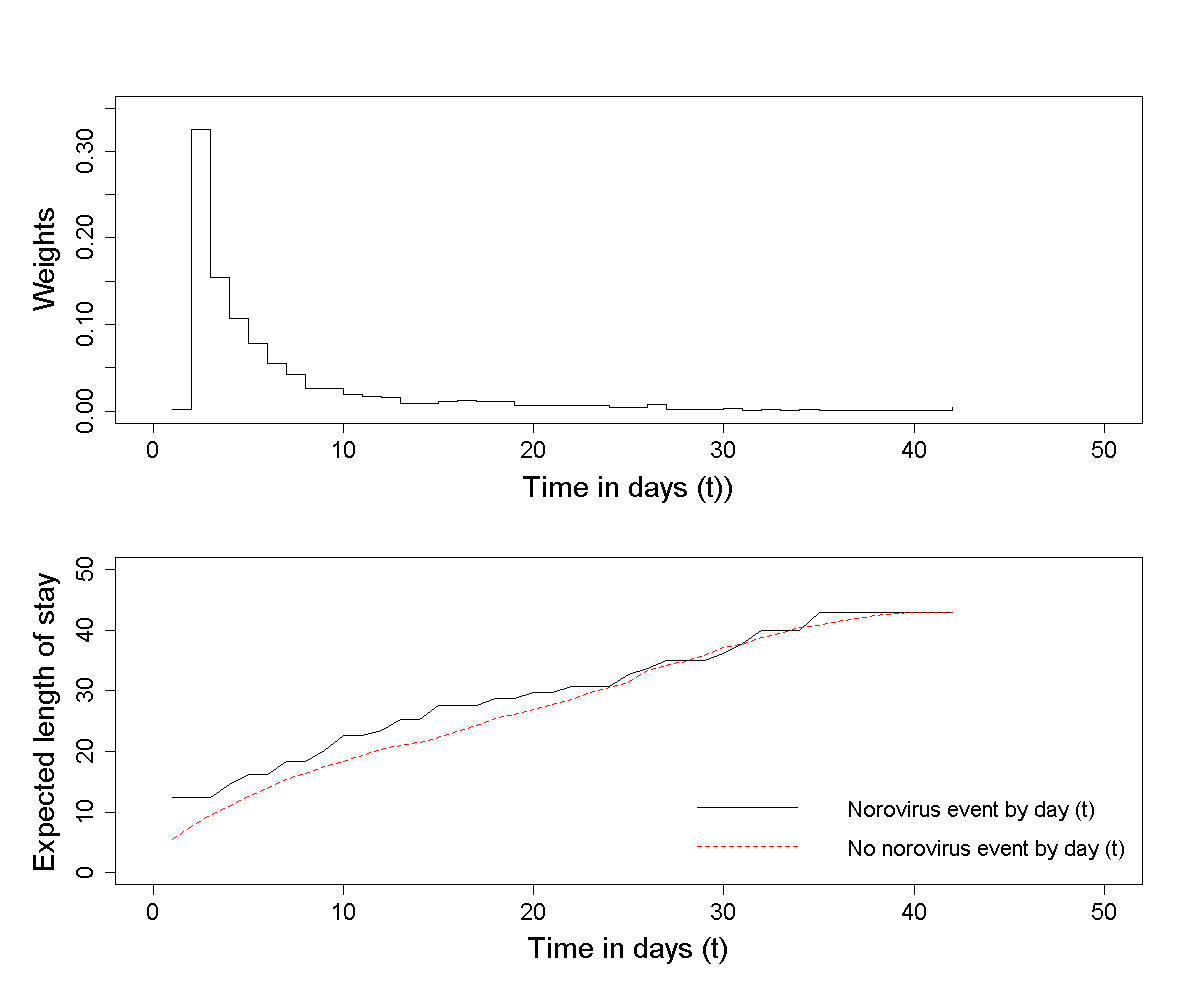
Figure 8. Frequency of developing norovirus, and the expected length of stay for norovirus and control patients. The upper panel shows how most inpatients developed norovirus during the first 10 days of their stay, while the lower panel shows the daily difference in the expected length of stay between norovirus and control patients, which remained relatively constant until around day 24.

Finally, we ran 10,000 bootstraps to obtain robust estimates of the standard error to calculate 95% confidence intervals. Although relatively wide, the obtained intervals corresponded to previously published durations of symptomatic disease in hospitalised norovirus patients of 1 to 8 days [19-22]. Moreover, the negative lower-bound confidence interval obtained for norovirus cases with primary diagnoses points towards them having left the model faster than control patients (i.e., hazard rate λ_02_ < λ_12_; cf. Supplementary Figure 6), which may be explained by a short overall length of stay and fast discharge of norovirus cases when admitted for acute gastrointestinal symptoms only. Another possible explanation would be a higher in-hospital mortality of norovirus cases (i.e., λ_03_ < λ_13_) [16], which was not applicable in our sample.

1. Details on the comparison of bed-days kept unoccupied for infection control

For a fair comparison of the bed-days kept unoccupied due to norovirus, we matched the figures voluntarily reported to HNORS during norovirus outbreaks with those mandatorily recorded by NHS England for acute care hospitals during winters. We used the resulting ratio to scale up figures in HNORS.

For HNORS, we limited outbreaks to those of acute care hospitals, excluded maternity and mental health wards, and restricted outbreaks to the same range of dates recorded each winter by NHS England via the start and end date of outbreaks. Thus, of the 8,142 outbreaks included in our study during July 2009 to June 2016, we first excluded 39 outbreaks in community and mental health hospitals as well as 41 outbreaks in maternity and mental health wards. Limiting the outbreaks to the same days during winters as recorded by NHS England excluded 4,803 outbreaks, of which n=1,756 belonged to the entire season 2009/10 given that NHS England started recording only in winter 2010/11. In total, 3,259 outbreaks remained for the comparison that made up for 40.4% of all outbreaks in HNORS, while the bed-days lost in those outbreaks made up for about 52% of all bed-days lost in HNORS. Figure 9 illustrates the difference between the outbreaks reported annually and the outbreaks considered during the winters of 2010/11 to 2015/16.


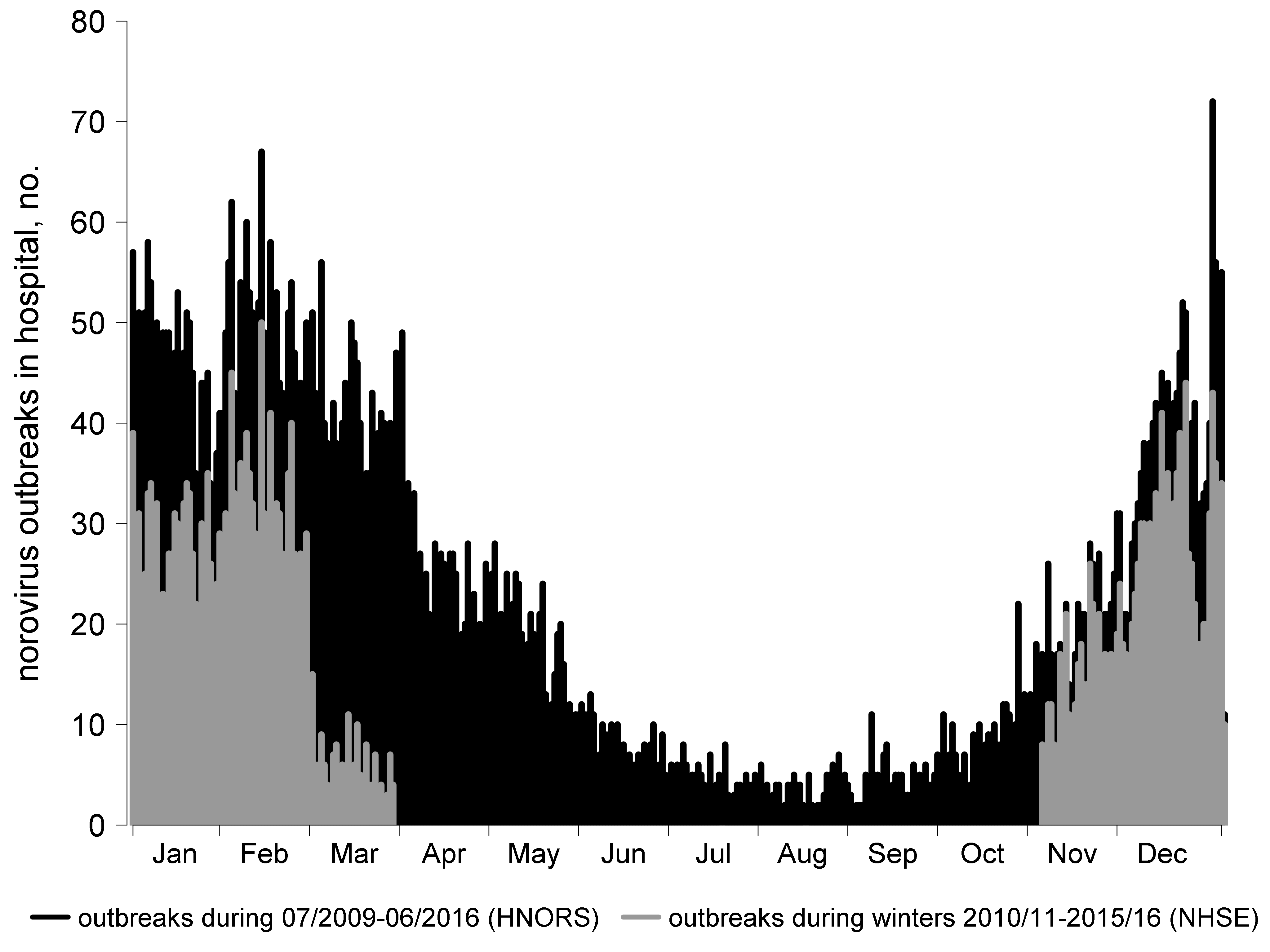
Figure 9. Daily number of outbreaks recorded in HNORS between July 2009 to June 2016, and of outbreaks during the same period of time as recorded by NHS England, per start date.

In addition, we explored whether the bed-days lost recorded in HNORS were associated with norovirus by repeating the linear regression described above. Norovirus was the only covariate that was non-negative and statistically significant for predicting the number of bed-days lost in outbreaks. The adjusted R^2^ is suggestive of norovirus laboratory reports being able to explain more than 80% of the bed-days lost (Supplementary Figure 10).

Figure 10. Weekly number of bed-days lost recorded in HNORS between July 2009 to June 2016.


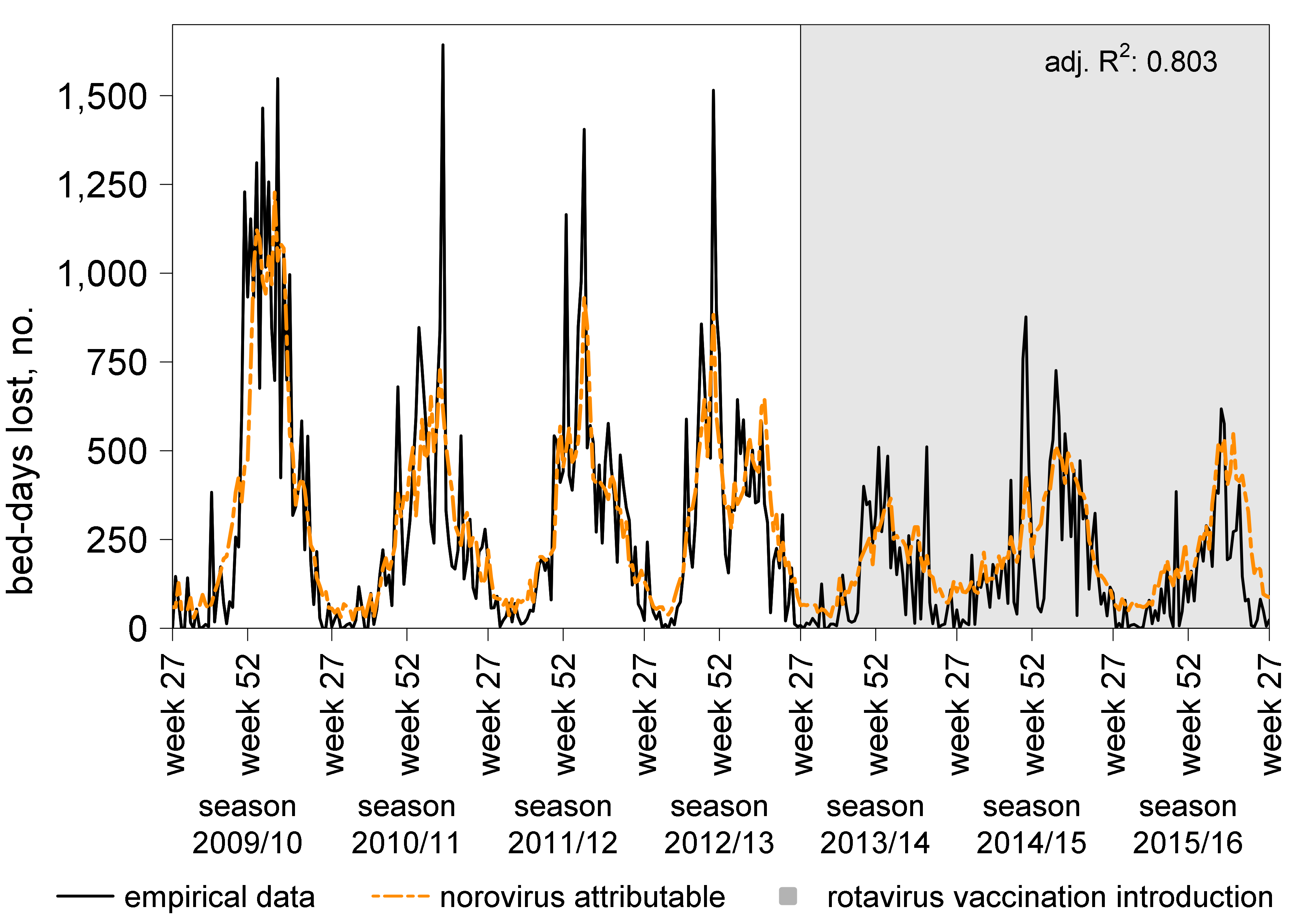


For NHS England, we imputed missing values for weekends and Christmas holidays in best-to-worst-case scenarios (i.e., lowest to highest imputations) [4]. Afterwards, we approximated outbreaks using conventional definitions for norovirus [19, 23, 24] by excluding all single beds that were unavailable for merely one day within 48 hours, which reduced the 142,100–186,000 bed-days across all six winters with the lowest-to-highest imputations only marginally to 141,600–185,800 bed-days.

We also investigated whether unavailable beds recorded by NHS England were associated with norovirus by repeating the linear regression described above. Norovirus was always the only covariate left in all regression models (including scenario analyses) that was non-negative and statistically significant for predicting the hospital bed-days closed unoccupied, occupied, and combined for both the lowest and highest imputations (Supplementary Figure 11). When calculating Pearson’s correlation coefficient to investigate the linear relationship between the number of bed-days lost during outbreaks (in HNORS) and the unoccupied bed-days unavailable (by NHS England) during winters per week, we found positive correlations of *r*=0.76 for the lowest imputations and *r*=0.73 for the highest imputations.


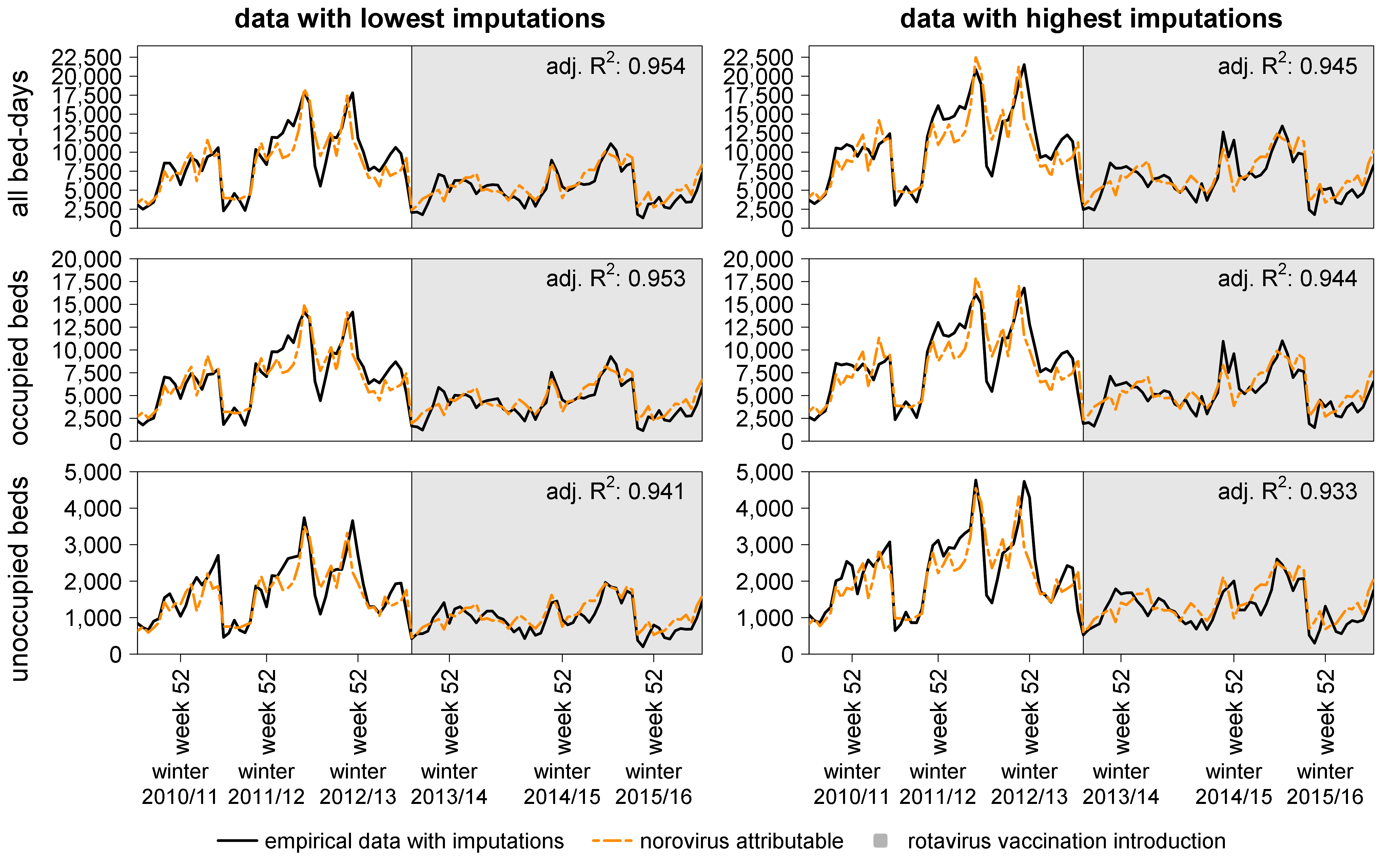
Figure 11. Weekly number of bed-days closed due to diarrhoea and vomiting/norovirus-like symptoms recorded by NHS England during winters, 2010/11 to 2015/16. Note the different scales for the horizontal planes.

Overall, 21.2%–28.0% of unoccupied bed-days recorded by NHS England matched the bed-days reported for outbreaks to HNORS (Supplementary Figure 12). Between July 2009 and June 2013, the number of matching bed-days was higher with 22.6%–29.7%, which decreased in subsequent years to 19.7%–26.3% between July 2013 and June 2016. Apart from the mode of reporting (i.e., voluntary to HNORS vs. mandatory to NHS England), other possible explanations for this discrepancy could be that hospitals start reporting an outbreak to HNORS but do not update their report once the outbreak has finished, which would thus not contain all numbers of bed-days lost, staff absences and patients involved. Also, hospitals may perceive both systems as duplicate; or there may be a lack of general awareness of HNORS.


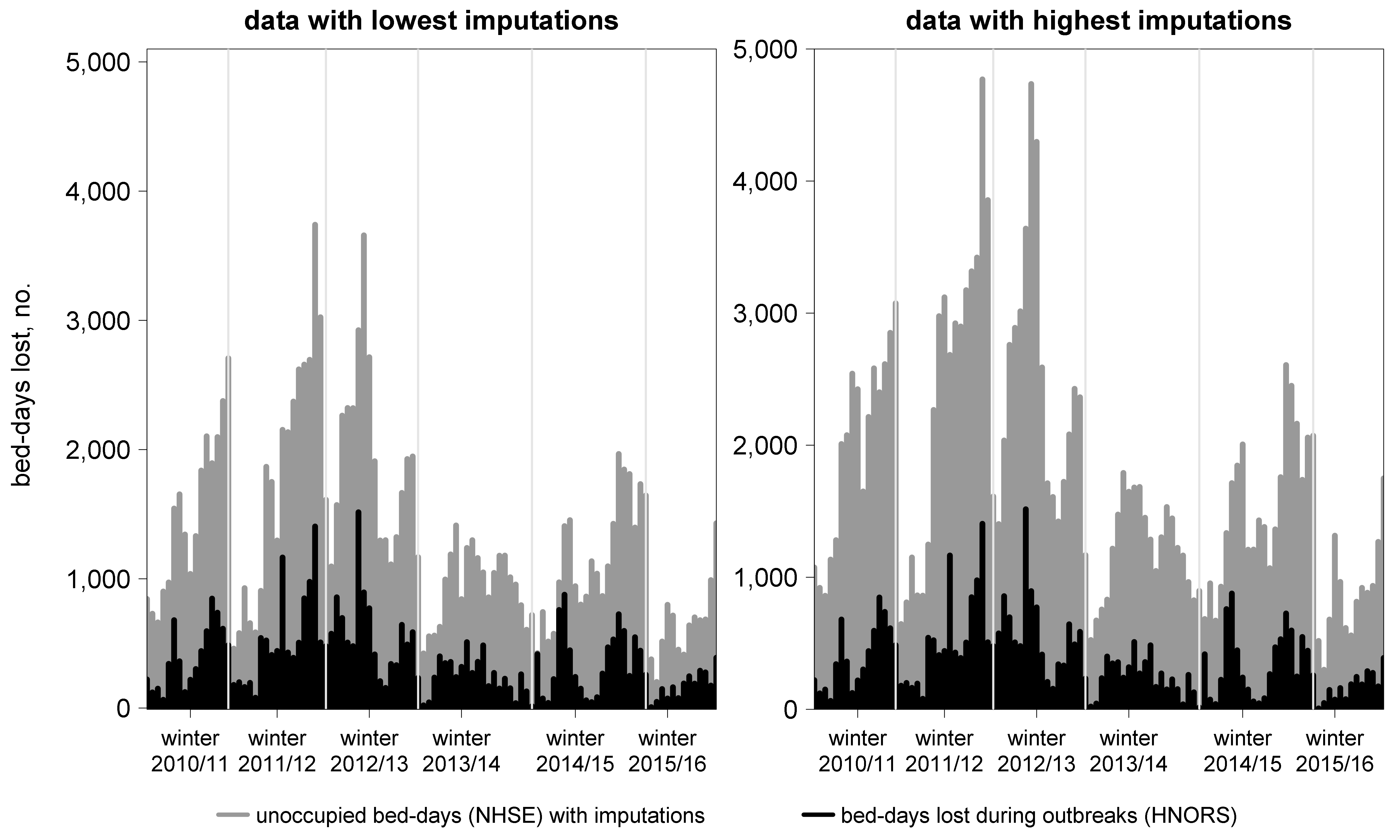
Figure 12. Matched weekly number of bed-days lost during outbreaks reported to HNORS vs. lowest and highest imputations of unoccupied bed-days recorded by NHS England, winters 2010/11 to 2015/16.

Assuming that the observed difference applied throughout the year and also to community hospitals and maternity and mental health wards, we scaled up the numbers of bed-days lost recorded in HNORS (Supplementary Table 9). For 2009/10, we used the figures for across the other six winters given that NHS England only started recording the winter situation in 2010/11. Note that adjusting for potential under-reporting of outbreaks explicitly first before scaling up the then-higher figures of bed-days with the then-lower number of non-matching bed-days would give the same results.

| Table 9: National trend of hospital bed-days lost unoccupied in England per winter, 2009/10–2015/16 | | | | | | | |
| --- | --- | --- | --- | --- | --- | --- | --- |
| **Source** | **2009/10** | **2010/11** | **2011/12** | **2012/13** | **2013/14** | **2014/15** | **2015/16** |
| **HNORS^a^** | n/a | 6,300 | 9,400 | 9,700 | 5,000 | 7,500 | 2,300 |
| **NHSE^b^** | n/a | 24,000–31,700 | 32,000–42,600 | 32,500–41,900 | 19,700–25,400 | 24,700–32,600 | 8,600–11,500 |
| **matching (%)** | n/a | 19.4–25.2 | 22.6–30.1 | 23.4–29.7 | 19.4–25.3 | 23.3–31.6 | 19.7–26.3 |
| **Adj. bed-days lost annually** | 81,700–108,000**^c^** | 60,700–78,600 | 57,300–76,300 | 57,000–72,400 | 29,200–38,000 | 39,100–53,100 | 27,400–36,400 |
| HNORS: Hospital Norovirus Outbreak Reporting System, n/a: not applicable, NHSE: National Health Service England  a: Limited to non-community hospitals, excluding maternity and mental health wards, and to the recording periods of NHSE each winter.  b: Imputed missing values for weekends and Christmas holidays in best-to-worst-case scenarios, excluded isolated beds closed without another bed closed in 48 hours in order to approximate outbreaks, and ensured association with norovirus via linear regressions.  c: Given that NHS England started recording in winter 2010/11, we used the six other winters to adjust the number for 2009/10. | | | | | | | |

1. Details on the estimated number of staff being absent during norovirus outbreaks

Potential under-reporting of the number of staff being absent during outbreaks were accounted for by multiplying the number of patients reported to HNORS with the ratio of infected patients to staff of 1:0.63 from a previous epidemiological study in one region of England that closely monitored norovirus outbreaks during April 2002 and March 2003, involving 2,154 patients and 1,360 healthcare staff [7]. Given that the number of patients involved in outbreaks is also under-recorded due to under-reporting of outbreaks [25], we first scaled patient figures up to 100%. Note: We did not take our estimated number of norovirus patients as baseline given that this will overestimate staff absences, which occur only for symptomatic disease and particularly during outbreaks.

Our adjustments led to figures that were about three times higher than the reported absences (Supplementary Table 10). Overall, there were an estimated median 9,100 (IQR: 5,100–12,000) individual staff absences during outbreaks annually between July 2009 and June 2016.

| Table 10. Number of staff absences and patients during norovirus outbreaks in England per season. | | | | | | | |
| --- | --- | --- | --- | --- | --- | --- | --- |
| Variable | 2009/10 | 2010/11 | 2011/12 | 2012/13 | 2013/14 | 2014/15 | 2015/16 |
| Patients^a^ | 19,500 | 11,500 | 15,500 | 14,000 | 5,400 | 7,700 | 4,300 |
| staff absences^a^ | 5,200 | 3,000 | 3,700 | 3,500 | 1,400 | 2,100 | 1,300 |
| Patients^b^ | 24,400 | 14,400 | 19,400 | 17,500 | 6,700 | 9,600 | 5,300 |
| staff absences^c^ | 15,400 | 9,100 | 12,200 | 11,100 | 4,200 | 6,100 | 3,400 |
| HNORS: Hospital Norovirus Outbreak Reporting System.  a: Raw data of patients and staff absences from HNORS. Raw staff absences only shown for reference here.  b: Scaled up to 100% to account for under-reporting of outbreaks [25].  c: Estimated by multiplying the number of patients with the ratio of patients to staff from a previous norovirus outbreak study in England [7]. | | | | | | | |

Interestingly, there was a decrease in absences over time, which matched the decrease in reported outbreaks (Supplementary Table 3). Between July 2009 and June 2013 there were a median 12,000 (IQR: 11,000–13,000) individual staff absences, which decreased between July 2013 and June 2016 to only a median 4,200 (IQR: 3,800–5,100) staff absences. It is unclear whether this is a genuine decrease in recent seasons or a result of reporting bias, especially in light of the stable (if not slowly increasing) number of secondary norovirus-associated gastroenteritis diagnoses in England (Figure 1 in the main text).

1. Details of the costing approach

We costed inpatients with a primary norovirus diagnosis with the activity-weighted mean reference costs for elective, non-elective, non-elective short stay and day cases of gastrointestinal infections, with or without any intervention and with any Complication and Comorbidity (CC) score (i.e., all healthcare resource group codes starting with FZ36*) and paediatric infectious or non-infectious gastroenteritis, irrespective of the CC score (i.e., all codes starting with PF21*) [26].

For inpatients with secondary diagnoses attributable to norovirus, only their excess stay due to norovirus was costed by using the activity-weighted mean excess bed-day value for elective and non-elective stays of gastrointestinal infections (all codes starting with FZ36*) and paediatric infectious or non-infectious gastroenteritis, with any Complication and Comorbidity (CC) score (all codes starting with PF21*) [26]. We approximated the resource consumption of day cases with 0.5 bed-days.

For hospital bed-days kept unoccupied, we used the excess bed-day value for all elective and non-elective stays (all codes), irrespective of intervention and the CC score [26]. Excluding gastroenteritis would assume that all hospitalisations for gastroenteritis are unnecessary, which may not be true for the more severe forms.

Staff absences due to norovirus were costed using the average wage of the mid-range grade E of the NHS pay scale for nurses in England for 2015/16 [27]. We assumed an estimated 3.14 days of work missed per absence based on a previous norovirus outbreak study in England involving 1,360 staff members [7]. The estimated number of staff absences is likely to be still an underestimate due to considering only outbreaks and the average wage of nurses but no other healthcare professionals nor relief/locum staff. Other studies of norovirus outbreaks in England and Scotland applied the same assumptions, with their proportions of staff absence costs on the total expenditure reaching 0.25 [5] and 0.17 [9], while our estimated staff costs made up about 0.03.

For the next-best alternative patients forgone, whose forgone health benefit gain poses the relevant opportunity costs for decision makers aiming to maximise population health [28], we assumed that the national average of the regularly admitted non-gastroenteritis patients in England were a reasonable proxy [1, 26]. We costed these alternative patients with the activity-weighted mean reference costs for elective, non-elective, non-elective short stay and day cases of non-gastrointestinal diagnoses (excluding all codes starting with FZ36* or PF21*) [26].

The input parameters and the values used for multivariate sensitivity analyses are shown in Supplementary Table 11.

| Table 11: Input parameters of the calculations for bed-days. | | | |
| --- | --- | --- | --- |
| Parameter | **Value (sensitivity analysis)** | **Unit** | **Description and sources** |
| Length of stay | 5.01 (3.3, 7.2) | days | Mean hospital LOS of all non-gastroenteritis cases in England (HES, 2015/16). Sensitivity analysis: Lowest and highest mean LOS per non-gastroenteritis sub-group (local hospital sample). |
|  | 3.33 (0.17, 6.50) | days | Mean excess hospital LOS of patients with norovirus infection (local hospital sample). Sensitivity analysis: 95% confidence interval. |
| Unit costs | £1,072 (£800, £1,222) | per patient | Mean NHS reference costs of gastroenteritis cases in England in 2015/16 (activity-weighted, only HRGs FZ36 and PF21^a^) [26]. Sensitivity analysis: lower and upper quartiles. |
|  | £1,491 (£1,058, £1,766) | per patient | Mean NHS reference costs of non-gastroenteritis cases in England in 2015/16 (activity-weighted, excluding HRGs FZ36 and PF21^a^) [26]. Sensitivity analysis: lower and upper quartiles. |
|  | £294 (£232, £348) | per bed-day | Mean NHS reference costs of excess bed-days for gastroenteritis in England in 2015/16 (activity-weighted, only HRGs FZ36 and PF21^a^) [26]. Sensitivity analysis: lower and upper quartiles. |
|  | £306 (£220, £366) | per bed-day | Mean NHS reference costs of all excess bed-days in England in 2015/16 (activity-weighted, all HRGs) [26]. Sensitivity analysis: lower and upper quartiles. |
| Norovirus-attributable gastroenteritis | 0.124 (0.118, 0.133) | proportion | Estimated from cases with primary gastrointestinal diagnoses between July 2009 and June 2013. Sensitivity analysis: 95% confidence interval. |
|  | 0.177 (0.156, 0.216) | proportion | Estimated from cases with primary gastrointestinal diagnoses between July 2013 and June 2016. Sensitivity analysis: 95% confidence interval. |
|  | 0.202 (0.177, 0.247) | proportion | Estimated from cases with secondary gastrointestinal diagnoses between July 2009 and June 2013. Sensitivity analysis: 95% confidence interval. |
|  | 0.238 (0.206, 0.299) | proportion | Estimated from cases with secondary gastrointestinal diagnoses between July 2013 and June 2016. Sensitivity analysis: 95% confidence interval. |
| Variable costs | 0.15 (0.04, 0.34) | proportion | Estimated proportion of variable costs on total hospital costs [29]. Sensitivity analysis: range of proportions published for 16 medical specialties [29]. |
| QALY gain | 0.239 (0.142, 0.260) | per patient | Mean (discounted) QALYs gained from hospital treatment for non-gastroenteritis cases with chronic conditions (local hospital sample, n=871). Sensitivity analysis: Mean (discounted) QALYs gained of all patients without gastroenteritis (n=2,465) and with acute life-threatening conditions (n=537), respectively. Acute life-threatening conditions were included as extreme scenario only as it seemed unrealistic to assume forgoing them constantly. |
| Monetary value for QALYs | £20,000 (£13,000, £30,000) | per QALY | NICE reference case [30]. Sensitivity analysis: estimated from mortality data [31], and NICE’s upper-bound threshold [30]. |
| HES: hospital episode statistics, HRG: healthcare resource group, LOS: length of stay, NHS: National Health Service, NICE: National Institute for Health and Care Excellence, QALY: quality-adjusted life year.  a: HRG FZ36*: gastrointestinal infections; HRG PF21*: paediatric, infectious or non-infectious gastroenteritis | | | |

1. Details on the modelled health gain expected from hospital treatment

In the absence of national data on the health gain from hospital treatment, we estimated the expected mean quality-adjusted life years, QALYs, from individual patient-level data of a local teaching hospital in London, UK. We mapped mean age- and sex-specific health utilities for diseased individuals to the primary admission code [32], and we separated patients into three sub-groups based on all diagnosis codes and the Charlson comorbidity index [33] (Supplementary Figure 13):

1. For patients with acute life-threatening conditions (i.e., myocardial infarctions, congestive heart failures, and cerebrovascular diseases), we assumed immediate death without hospital treatment. With treatment, the health status was assumed to be stabilised and the utility score maintained until discharge, at which point the utility was assumed to gradually decline over the remaining age- and sex-specific life expectancy using life-tables for England [34].
2. For all other patients with chronic conditions (i.e., Charlson index > 0 but not acutely life-threatening), we assumed without treatment a steady decline of the health utility for the remainder of the life expectancy, while with treatment we made the same assumption as before of an initially stabilised health status before deterioration commences after discharge.
3. For patients without chronic or life-threatening conditions, we modelled a gradual recovery until discharge, while we assumed a delay in recovery without treatment lasting for the entire stay in hospital, after which patients make a natural recovery over the same period of time as with hospital treatment. We assumed no difference attributable to this hospital stay for the remaining life expectancy. For the recovered health status, we used utility norms published sex-specifically by Programme Budget Categories [31]. For 40 young patients, this non-age-specific utility norm was smaller than the age-specific diseased utility, for who we thus assumed a norm of 1. Alternatively, one could have assumed a utility norm value of 1 for the recovered health status in line with theory, which were to skew results upwards while ignoring the patients’ age, sex, and diseased utility (and thus dismissed by us).

For patients with chronic or life-threatening conditions we used the age- and sex-specific life expectancy from the Office for National Statistics [34]. For all cases of in-hospital mortality, QALYs were calculated using the observed survival time.


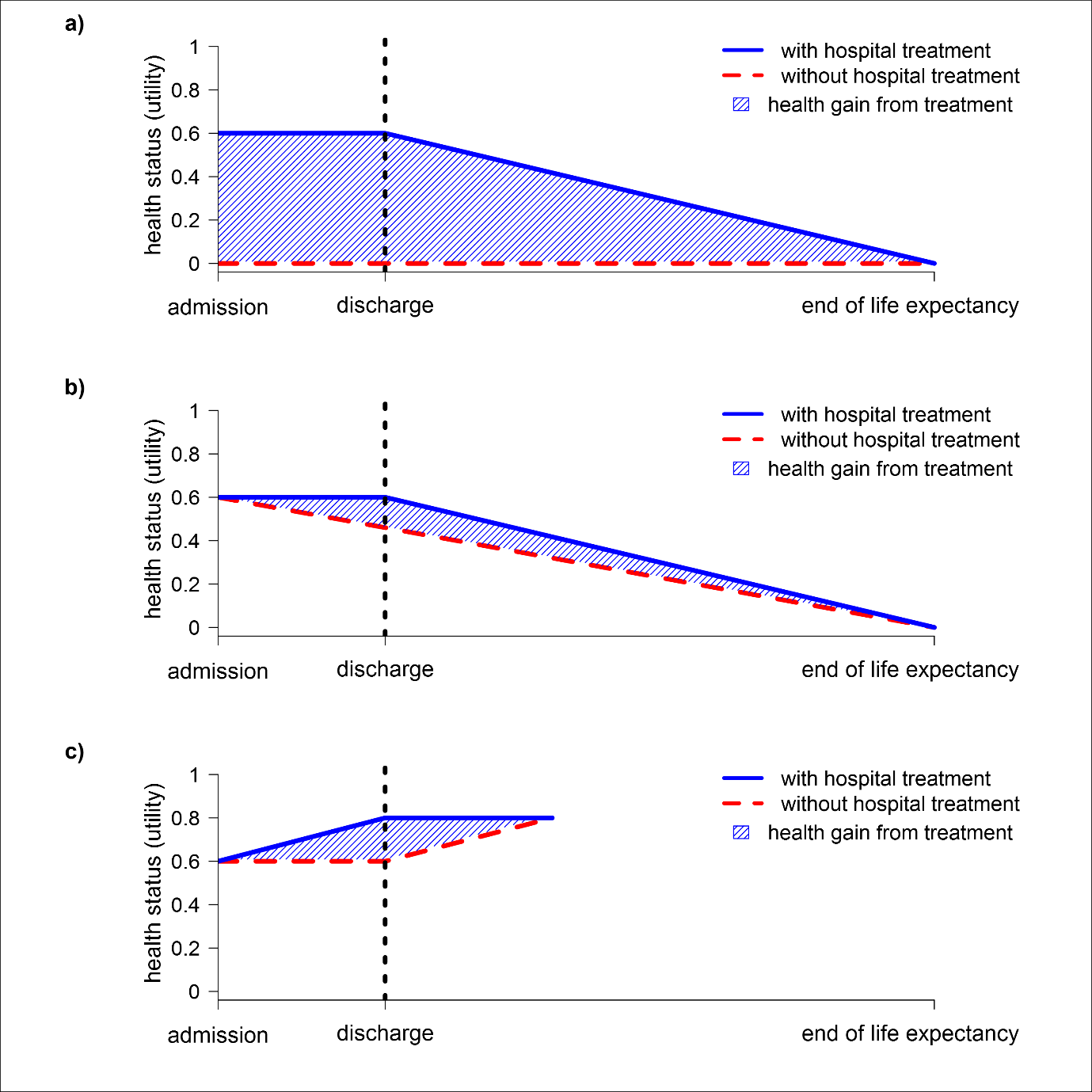
Figure 13. Health gain in terms of quality-adjusted life years, QALYs, from hospital treatment vs. no hospital treatment. To account for potential heterogeneity, the health gain modelling differed for the three patient sub-groups of a) acute life-threatening conditions, b) chronic conditions, or c) none of these conditions. a) Patients with acute life-threatening conditions survive with hospital treatment for their remaining age- and sex-specific life expectancy. b) Patients with chronic conditions maintain their health at a higher level with hospital treatment for their remaining age- and sex-specific life expectancy. c) Patients with none of these conditions recover faster with hospital treatment, but there is no effect attributable to this hospital stay for the remaining life expectancy.

1. Details on results of the burden and costs estimation

Table 12 shows the results of the burden estimation for patients, bed-days and staff absences per season.

| Table 12. Results for the burden of norovirus-associated gastroenteritis in hospital in England per season, 2009/10-2015/16 | | | | | | | |
| --- | --- | --- | --- | --- | --- | --- | --- |
| **Variable** | **2009/10** | **2010/11** | **2011/12** | **2012/13** | **2013/14** | **2014/15** | **2015/16** |
| **Patients** |  |  |  |  |  |  |  |
| ~ with primary norovirus diagnosis (95% CI)^a^ | 28,200  (26,800–30,200) | 27,300  (26,000–29,300) | 28,900  (27,500–31,000) | 30,700  (29,200–32,900) | 40,100  (35,300–48,900) | 41,900  (36,900–51,100) | 40,800  (36,000–49,800) |
| ~ with secondary norovirus diagnosis (95% CI)^b^ | 44,600  (39,100–54,600) | 44,300  (38,800–54,200) | 46,200  (40,500–56,500) | 49,100  (43,000–60,000) | 55,900  (48,400–70,200) | 61,500  (53,300–77,300) | 63,600  (55,000–79,800) |
| total (95% CI) | 72,800  (65,900–84,800) | 71,600  (64,800–83,500) | 75,100  (68,000–87,500) | 79,800  (72,200–92,900) | 96,000  (83,700–119,000) | 103,000  (90,200–128,000) | 104,000  (91,000–130,000) |
| **Bed-days** |  |  |  |  |  |  |  |
| ~ used for primary norovirus diagnoses (95% CI)^a^ | 70,500  (67,100–75,600) | 62,900  (59,900–67,500) | 61,400  (58,400–65,900) | 62,900  (59,900–67,500) | 81,400  (71,700–99,300) | 83,500  (73,600–102,000) | 76,800  (67,700–93,800) |
| ~ used for secondary norovirus diagnoses (95% CI)^c^ | 135,000  (118,000–165,000) | 132,000  (116,000–162,000) | 136,000  (119,000–167,000) | 146,000  (128,000–178,000) | 165,000  (143,000–207,000) | 181,000  (157,000–227,000) | 185,000  (160,000–233,000) |
| ~ lost unoccupied, lowest-to-highest imputations^d^ | 81,700–108,000 | 60,700–78,600 | 57,300–76,300 | 57,000–72,400 | 29,200–38,000 | 39,100–53,100 | 27,400–36,400 |
| total, low imputations (95% CI) | 287,000  (267,000–322,000) | 256,000  (236,000–290,000) | 255,000  (235,000–290,000) | 266,000  (245,000–303,000) | 275,000  (244,000–336,000) | 304,000  (269,000–368,000) | 290,000  (255,000–354,000) |
| total, high imputations (95% CI) | 313,000  (293,000–348,000) | 274,000  (254,000–308,000) | 274,000  (254,000–309,000) | 281,000  (260,000–318,000) | 284,000  (252,000–345,000) | 318,000  (283,000–382,000) | 299,000  (265,000–363,000) |
| **Staff absences due to illness** |  |  |  |  |  |  |  |
| total^e^ | 15,400 | 9,100 | 12,200 | 11,100 | 4,200 | 6,100 | 3,400 |
| HES: hospital episode statistics, HNORS: Hospital Norovirus Outbreak Reporting System, LOS: length of stay, NHS: National Health Service.  a: Derived from inpatients with a primary diagnosis of gastroenteritis attributed to norovirus with the two regression models with the highest goodness-of-fit for prior and after mid-2013.  b: Derived from inpatients with a secondary diagnosis of gastroenteritis attributed to norovirus with the two regression models with the highest goodness-of-fit for prior and after mid-2013.  c: Derived from the non-day cases with a norovirus-attributable secondary diagnosis of gastroenteritis times the estimated excess length of stay due to norovirus of 3.33 (95%-CI: 0.17–6.50) days plus the number of day cases with a norovirus-attributable secondary diagnosis of gastroenteritis times the approximated resource consumption of 0.5 bed-days.  d: Figures of bed-days lost were scaled up using the ratios derived from the comparison of bed-days during winters reported voluntarily to HNORS versus mandatorily to NHS England.  e: Figures account for under-reporting of outbreaks, and absences during outbreaks. | | | | | | | |

Table 13 shows the costing results, split up for the conventional costing and the opportunity costing of bed-days.

| Table 13. Results for the costs of norovirus-associated gastroenteritis in hospital in England per season, 2009/10-2015/16 | | | | | | | |
| --- | --- | --- | --- | --- | --- | --- | --- |
| **Variable** | **2009/10** | **2010/11** | **2011/12** | **2012/13** | **2013/14** | **2014/15** | **2015/16** |
| **Conventional costing** |  |  |  |  |  |  |  |
| Patients with primary norovirus diagnosis, in million £ (95% CI)^a^ | 30.2  (21.4–36.9) | 29.3  (20.8–35.8) | 31.0  (22.0–37.9) | 32.9  (23.4–40.2) | 43.0  (28.3–59.7) | 44.9  (29.5–62.4) | 43.8  (28.8–60.9) |
| Bed-days used for secondary norovirus diagnoses, in million £ (95% CI)^b^ | 39.5  (27.3–57.4) | 38.8  (26.8–56.3) | 40.0  (27.7–58.0) | 42.8  (29.6–62.1) | 48.4  (33.1–72.1) | 53.2  (36.3–79.2) | 54.4  (37.1–81.1) |
| Bed-days lost unoccupied, low-to-high imputations, in million £^c^ | 25.0–33.0 | 18.6–24.0 | 17.5–23.3 | 17.4–22.1 | 8.9–11.6 | 12.0–16.2 | 8.4–11.1 |
| Staff absence costs due to illness, in million £^d^ | 4.9 | 2.9 | 3.9 | 3.5 | 1.3 | 1.9 | 1.1 |
| Total, low-to-high imputations, in million £ | 99.6–107.6 | 89.5–95.0 | 92.4–98.2 | 96.7–101.4 | 101.6–104.3 | 111.9–116.2 | 107.6–110.4 |
| **Opportunity costing** |  |  |  |  |  |  |  |
| Patients forgone, low-to-high | 57,300–62,500 | 51,100–54,600 | 50,900–54,700 | 53,100–56,100 | 55,000–56,700 | 60,600–63,400 | 57,800–59,600 |
| QALYs forgone, low-to-high | 13,700–14,900 | 12,200–13,100 | 12,200–13,100 | 12,700–13,400 | 13,100–13,600 | 14,500–15,200 | 13,800–14,200 |
| Net monetary benefit, low-to-high, in million £^e^ | 188.4–205.7 | 168.0–179.7 | 167.4–179.9 | 174.6–184.7 | 180.9–186.7 | 199.4–208.6 | 190.1–196.1 |
| HES: hospital episode statistics, HNORS: Hospital Norovirus Outbreak Reporting System, LOS: length of stay, NHS: National Health Service.  a: Patients with primary diagnoses were costed directly using NHS reference costs [26].  b: Patients with secondary diagnoses were not costed directly given that they were in hospital for other primary reasons. Instead, we costed their excess bed-days due to norovirus.  c: Figures of bed-days lost were scaled up using the ratios derived from the comparison of bed-days reported during winters to HNORS vs. NHS England.  d: Figures account for under-reporting of outbreaks, and absences during outbreaks.  e: Equivalent to opportunity costs, unless a higher net benefit was achievable with the alternative patients forgone than with the norovirus patients. | | | | | | | |

The net monetary benefit of the QALYs forgone is equivalent to the opportunity costs of the norovirus patients, unless a higher net benefit was achievable with the alternative patients forgone [28]. In order to investigate this, we costed the QALY gain of norovirus patients with a primary diagnosis (0.078; Table 1) and a secondary norovirus diagnoses, for which we approximated the gastroenteritis-related health gain by subtracting the QALY gain of control patients with no primary gastroenteritis diagnosis and no norovirus infection from the QALY gain of inpatients with no primary gastroenteritis diagnosis but norovirus infection (i.e., 0.211-0.142=0.069; cf. Table 1 and Supplementary Table 4). The results of this analysis are shown in Table 14, which estimated that norovirus patients gained from hospital treatment an expected number of 5,400 QALYs before July 2013 and 7,500 QALYs after July 2013.

| Table 14. Costing results for the bed-days used for norovirus-associated inpatients in England, 2009/10-2015/16 | | | | | | | | |
| --- | --- | --- | --- | --- | --- | --- | --- | --- |
|  | Before July 2013:  261,000 bed-days | |  | After July 2013:  290,000 bed-days | |  | Across seasons:  275,000 bed-days | |
|  | Norovirus cases,  n=73,900 | Forgone patients:  n=52,100 |  | Norovirus cases,  n=103,400 | Forgone patients:  n=57,800 |  | Norovirus cases,  n=79,800 | Forgone patients:  n=55,000 |
| Expenditure  (£ million in total) | 94.5 | 77.6 |  | 107.6 | 86.1 |  | 99.6 | 82.0 |
| Benefit (GMB, £ million in total) | 107.2 | 248.9 |  | 150.2 | 276.2 |  | 115.6 | 262.8 |
| Net benefit (NMB, £ million in total) | 12.7 | 171.3 |  | 42.6 | 190.1 |  | 16.0 | 180.9 |
| Economic costs  (£ million in total)^a^ | 265.5 | 90.3 |  | 297.7 | 128.7 |  | 282.5 | 98.0 |
| GMB: gross monetary benefit (i.e., QALYs gained times £20,000 [30]), NICE: National Institute for Health and Care Excellence, NMB: net monetary benefit (i.e., benefit-expenditure), QALY: quality-adjusted life year.  a: Economic costs are defined as the expenditure incurred plus the highest net monetary benefit forgone; they approximate to opportunity costs in case the chosen option was the sub-optimal alternative. | | | | | | | | |

The net monetary benefit of the forgone patients was always higher than that of norovirus patients, and the economic costs were always lower than the benefit for norovirus only (Supplementary Table 14). As such, the higher net benefit was achievable with the forgone patients, rendering the norovirus cases as sub-optimal treatment choice from an economic perspective aiming to maximise population health. Consequently, the economic costs approximate to opportunity costs here.

1. Details on the sensitivity analysis

For the burden estimation, we used the estimated confidence intervals of our best-fitting regression models to obtain lower and upper estimates for the norovirus-attributable cases and the bed-days of primary diagnoses, and we used the estimated confidence interval of the excess length of stay due to norovirus from the multi-state model. Due to the wide confidence interval of the excess hospital stay of norovirus cases, the bed-days used for them showed the highest uncertainty to either side of the base case value (Supplementary Figure 14). For the bed-days lost unoccupied for infection control, we took the lower value as conservative estimate, which is why there is only an upwards trend shown (Supplementary Figure 14); the actual number of bed-days kept unoccupied is likely higher than we assumed but also lower than the worst-case scenario.


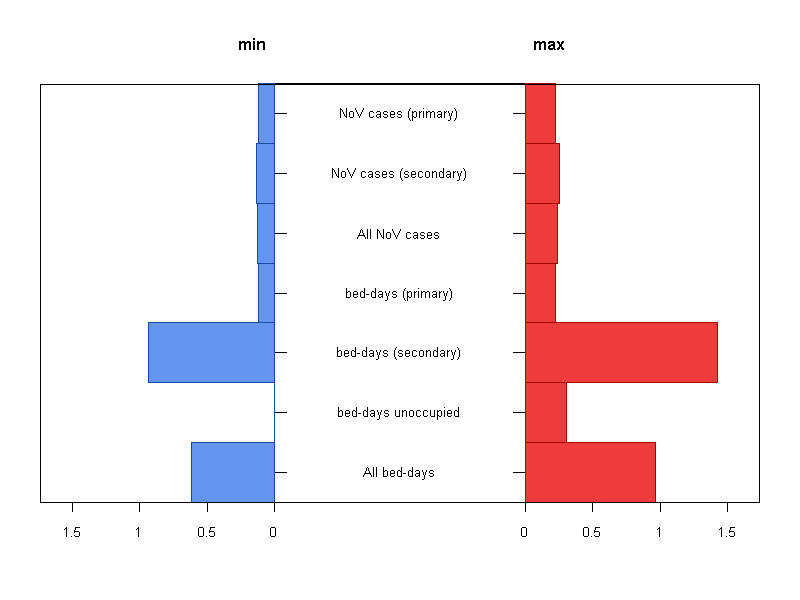
Figure 14. Tornado diagram of the change (in %) of the base estimates for the burden estimation. NoV: norovirus.

For the costs, estimates appeared to be right-skewed, as is often the case for cost data [35]. There appeared to be less uncertainty surrounding calculations using the length of stay and expenditure of the forgone alternative patients (Supplementary Figure 15: “nr forgone pts”, “costs forgone pts”) than for calculations relying on a monetary value for the quality-adjusted life years, QALYs, gained (Supplementary Figure 15, “GMB forgone pts”, “NMB forgone pts”, “economic costs NoV”). For the variable costs proportion of the expenditure on norovirus (“variable costs NoV”), the wide interval chosen as input of up to 0.34 is reflecting the large uncertainty surrounding the estimate when the total expenditures also increased.


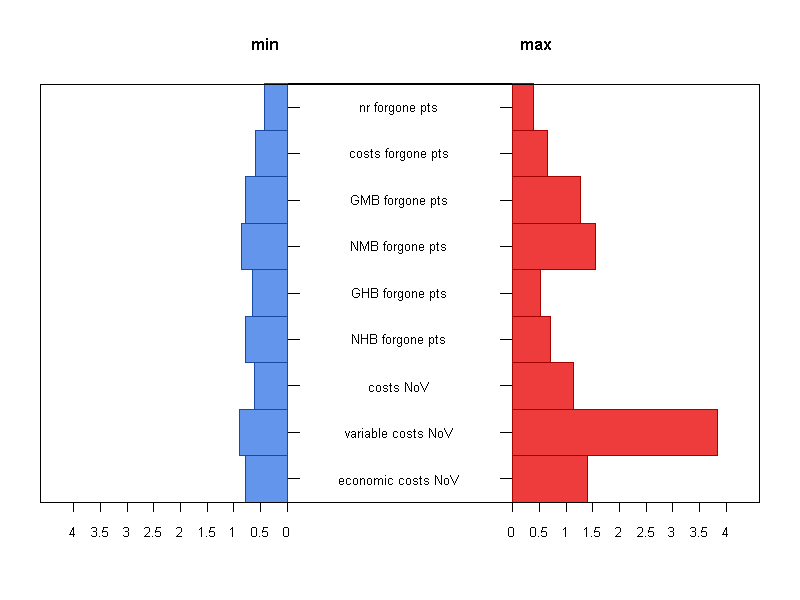


Figure 15. Tornado diagram of the change (in %) of the base estimates for the cost calculation. GHB: gross health benefit, GMB: gross monetary benefit, NHB: net health benefit, NMB: net monetary benefit, NoV: norovirus, pts: patients.

1. References

1. Health and Social Care Information Centre. Hospital Admitted Patient Care Activity, 2015-16. Available at: http://www.content.digital.nhs.uk/catalogue/PUB22378/hosp-epis-stat-admi-summ-rep-2015-16-rep.pdf.

2. Public Health England. Rotavirus vaccination programme for infants. Available at: https://www.gov.uk/government/collections/rotavirus-vaccination-progarmme-for-infants. Accessed July 2016.

3. Public Health England. Norovirus and rotavirus: summary of surveillance. Available at: https://www.gov.uk/government/statistics/norovirus-national-update. Accessed 26/01/2017.

4. Sandmann FG, Jit M, Robotham JV, Deeny SR. Burden, duration, and costs of hospital bed closures due to acute gastroenteritis in England per winter, 2010/11–2015/16. The Journal of hospital infection **2017**.

5. Atchison CJ, Lopman BA, Harris CJ, Tam CC, Iturriza Gomara M, Gray JJ. Clinical laboratory practices for the detection of rotavirus in England and Wales: can surveillance based on routine laboratory testing data be used to evaluate the impact of vaccination? Euro Surveill **2009**; 14(20).

6. Kambhampati A, Koopmans M, Lopman B. Burden of norovirus in healthcare facilities and strategies for outbreak control. Journal of Hospital Infection **2015**; 89(4): 296-301.

7. Lopman BA, Reacher MH, Vipond IB, et al. Epidemiology and cost of nosocomial gastroenteritis, Avon, England, 2002-2003. Emerging infectious diseases **2004**; 10(10): 1827-34.

8. Ryan MJ, Ramsay M, Brown D, Gay NJ, Farrington CP, Wall PG. Hospital admissions attributable to rotavirus infection in England and Wales. J Infect Dis **1996**; 174 Suppl 1: S12-8.

9. Harris JP, Jit M, Cooper D, Edmunds WJ. Evaluating rotavirus vaccination in England and Wales. Part I. Estimating the burden of disease. Vaccine **2007**; 25(20): 3962-70.

10. Haustein T, Harris JP, Pebody R, Lopman BA. Hospital admissions due to norovirus in adult and elderly patients in England. Clinical infectious diseases : an official publication of the Infectious Diseases Society of America **2009**; 49(12): 1890-2.

11. Atchison CJ, Stowe J, Andrews N, et al. Rapid Declines in Age Group-Specific Rotavirus Infection and Acute Gastroenteritis Among Vaccinated and Unvaccinated Individuals Within 1 Year of Rotavirus Vaccine Introduction in England and Wales. J Infect Dis **2016**; 213(2): 243-9.

12. Thomas SL, Walker JL, Fenty J, et al. Impact of the national rotavirus vaccination programme on acute gastroenteritis in England and associated costs averted. Vaccine **2017**; 35(4): 680-6.

13. Allen DJ, Adams NL, Aladin F, Harris JP, Brown DW. Emergence of the GII-4 Norovirus Sydney2012 strain in England, winter 2012-2013. PloS one **2014**; 9(2): e88978.

14. De Angelis G, Murthy A, Beyersmann J, Harbarth S. Estimating the impact of healthcare-associated infections on length of stay and costs. Clinical microbiology and infection : the official publication of the European Society of Clinical Microbiology and Infectious Diseases **2010**; 16(12): 1729-35.

15. Beyersmann J, Gastmeier P, Wolkewitz M, Schumacher M. An easy mathematical proof showed that time-dependent bias inevitably leads to biased effect estimation. J Clin Epidemiol **2008**; 61(12): 1216-21.

16. Barnett AG, Beyersmann J, Allignol A, Rosenthal VD, Graves N, Wolkewitz M. The time-dependent bias and its effect on extra length of stay due to nosocomial infection. Value Health **2011**; 14(2): 381-6.

17. Harris JP, Edmunds WJ, Pebody R, Brown DW, Lopman BA. Deaths from norovirus among the elderly, England and Wales. Emerging infectious diseases **2008**; 14(10): 1546.

18. Allignol A, Schumacher M, Beyersmann J. Empirical transition matrix of multi-state models: the etm package. Journal of Statistical Software **2011**; 38(4): 1-15.

19. Lopman BA, Reacher MH, Vipond IB, Sarangi J, Brown DW. Clinical manifestation of norovirus gastroenteritis in health care settings. Clinical infectious diseases : an official publication of the Infectious Diseases Society of America **2004**; 39(3): 318-24.

20. Patel MM, Hall AJ, Vinjé J, Parashar UD. Noroviruses: a comprehensive review. Journal of Clinical Virology **2009**; 44(1): 1-8.

21. Lee BY, McGlone SM, Bailey RR, Wettstein ZS, Umscheid CA, Muder RR. Economic impact of outbreaks of norovirus infection in hospitals. Infection Control and Hospital Epidemiology **2011**; 32(2): 191.

22. Bartsch SM, Huang SS, Wong KF, Avery TR, Lee BY. The spread and control of norovirus outbreaks among hospitals in a region: a simulation model. Open forum infectious diseases **2014**; 1(2): ofu030.

23. Chadwick PR, Beards G, Brown D, et al. Management of hospital outbreaks of gastro-enteritis due to small roundstructured viruses. The Journal of hospital infection **2000**; 45(1): 1-10.

24. Harris JP, Lopman BA, Cooper BS, O'Brien SJ. Does spatial proximity drive norovirus transmission during outbreaks in hospitals? BMJ open **2013**; 3(7).

25. Harris JP, Adams NL, Lopman BA, Allen DJ, Adak GK. The development of Web-based surveillance provides new insights into the burden of norovirus outbreaks in hospitals in England. Epidemiology and infection **2014**; 142(8): 1590-8.

26. Department of Health. NHS reference costs 2015 to 2016. Available at: https://www.gov.uk/government/publications/nhs-reference-costs-2015-to-2016. Accessed 04/02/2017.

27. Royal College of Nursing. NHS pay scales 2015-16: Pay scales for NHS nursing staff in England, Wales, Scotland and Northern Ireland from 1 April 2015. Available at: https://www.rcn.org.uk/employment-and-pay/nhs-pay-scales-2015-16. Accessed 03/02/2017.

28. Sandmann FG, Robotham JV, Deeny SR, Edmunds WJ, Jit M. Estimating the opportunity costs of bed-days. Health Econ **2017**

29. Plowman R, Graves N, Griffin MA, et al. The rate and cost of hospital-acquired infections occurring in patients admitted to selected specialties of a district general hospital in England and the national burden imposed. The Journal of hospital infection **2001**; 47(3): 198-209.

30. NICE. Guide to the methods of technology appraisal 2013. London: National Institute for Health and Care Excellence, **2013**.

31. Claxton K, Martin S, Soares M, et al. Methods for the estimation of the National Institute for Health and Care Excellence cost-effectiveness threshold. Health technology assessment **2015**; 19(14): 1-503, v-vi.

32. Claxton K, Martin S, Soares M, et al. Appendix A (Displacement by ICD code). Available at: https://www.york.ac.uk/che/research/teehta/thresholds/. Accessed 04/02/2017.

33. Quan H, Sundararajan V, Halfon P, et al. Coding algorithms for defining comorbidities in ICD-9-CM and ICD-10 administrative data. Medical care **2005**; 43(11): 1130-9.

34. Office for National Statistics. National life tables, UK Statistical bulletins. Available at: https://www.ons.gov.uk/peoplepopulationandcommunity/birthsdeathsandmarriages/lifeexpectancies/bulletins/nationallifetablesunitedkingdom/previousReleases. Accessed 04/02/2017.

35. Drummond MF, Sculpher MJ, Torrance GW, O'Brien BJ, Stoddart GL. Methods for the economic evaluation of health care programme. Third edition: Oxford: Oxford University Press, **2005**.
